# Distinct phosphorylation sites in a prototypical GPCR differently orchestrate β-arrestin interaction, trafficking, and signaling

**DOI:** 10.1126/sciadv.abb8368

**Published:** 2020-09-11

**Authors:** Hemlata Dwivedi-Agnihotri, Madhu Chaturvedi, Mithu Baidya, Tomasz Maciej Stepniewski, Shubhi Pandey, Jagannath Maharana, Ashish Srivastava, Natarin Caengprasath, Aylin C. Hanyaloglu, Jana Selent, Arun K. Shukla

**Affiliations:** 1 Department of Biological Sciences and Bioengineering, Indian Institute of Technology, Kanpur 208016, India.; 2 Research Programme on Biomedical Informatics (GRIB), Department of Experimental and Health Sciences of Pompeu Fabra University (UPF)-Hospital del Mar Medical Research Institute (IMIM), 08003 Barcelona, Spain.; 3 Faculty of Chemistry, Biological and Chemical Research Centre, University of Warsaw, Warsaw, Poland.; 4 Institute of Reproductive and Developmental Biology, Department of Metabolism, Digestion and Reproduction, Hammersmith Campus, Imperial College London, Du Cane Road, London, W12 0NN, UK.

## Abstract

Agonist-induced phosphorylation of G protein–coupled receptors (GPCRs) is a key determinant for their interaction with β-arrestins (βarrs) and subsequent functional responses. Therefore, it is important to decipher the contribution and interplay of different receptor phosphorylation sites in governing βarr interaction and functional outcomes. Here, we find that several phosphorylation sites in the human vasopressin receptor (V_2_R), positioned either individually or in clusters, differentially contribute to βarr recruitment, trafficking, and ERK1/2 activation. Even a single phosphorylation site in V_2_R, suitably positioned to cross-talk with a key residue in βarrs, has a decisive contribution in βarr recruitment, and its mutation results in strong G-protein bias. Molecular dynamics simulation provides mechanistic insights into the pivotal role of this key phosphorylation site in governing the stability of βarr interaction and regulating the interdomain rotation in βarrs. Our findings uncover important structural aspects to better understand the framework of GPCR-βarr interaction and biased signaling.

## Teaser

GPCR–β-arrestin interaction and signaling are differentially regulated by distinct phosphorylation sites on the receptor.

## INTRODUCTION

The interaction of β-arrestins (βarrs) with G protein–coupled receptors (GPCRs) is a versatile mechanism to regulate agonist-induced downstream signaling and trafficking of these receptors (*1*–*3*). In addition to their well-established contribution in terminating G-protein signaling and driving activated receptors to endocytic routes, βarrs are now also appreciated to facilitate the formation of receptor–G-protein–βarr megaplexes (*4*, *5*). Furthermore, βarrs also contribute positively toward downstream signaling cascades such as activation of MAP kinases, although a complete G-protein dependence of this phenomenon is currently discussed and debated (*2*, *6*–*9*). The recruitment of βarrs involves two distinct but interlinked features of GPCRs, namely, agonist-induced receptor activation and receptor phosphorylation, which engage different interfaces on βarrs (*10*, *11*). Recent studies have demonstrated an appreciable level of functional distinction associated with the two sets of interactions between GPCRs and βarrs, i.e., through the receptor core and phosphorylated C terminus and resulting conformations of GPCR-βarr complexes (*12*–*14*).

On the basis of the temporal stability of their interaction with βarrs and trafficking patterns, GPCRs are typically categorized into two broad classes referred to as class A and B (*15*). While class A GPCRs have transient interaction with βarrs resulting in rapid recycling, class B GPCRs exhibit a relatively stable and sustained interaction leading to their slow recycling and proteosomal degradation (*15*, *16*). Cumulative phosphorylation of GPCRs, especially in clusters of serine and threonine residues, is typically conceived to determine the stability of βarr binding (*15*, *17*). A recent study has also proposed the presence or absence, and relative frequencies, of specific phosphorylation codes in the receptors as an important determinant of the stability patterns of GPCR-βarr interaction (*18*). In addition, it is also established that specific phosphorylation patterns in GPCRs arising from phosphorylation by different kinases can drive distinct βarr conformations leading to different functional outcomes, a framework that is referred to as phosphorylation “barcode” (*19*, *20*).

While these studies have collectively established the current conceptual framework of GPCR-βarr interaction, a clear structural understanding of how specific receptor phosphorylation sites are linked to βarr recruitment, activation, and conformational changes still remains relatively less well understood. A key limitation until recently has been the lack of structural templates of GPCR-βarr complexes to design structure-guided systematic strategies, to probe and directly correlate the contribution of specific phosphorylation sites in βarr recruitment and functional outcomes. However, there has been a notable progress on direct structural visualization of GPCR-arrestin interaction over the last few years using x-ray crystallography and cryo–electron microscopy (*11*, *18*, *21*–*25*). These advances now allow structure-guided experimental design and interpretation of data to better understand the intricate details of GPCR-βarr interaction and their functional relevance.

In this study, we set out to probe the contribution of different phosphorylation sites in the human vasopressin receptor (V_2_R), a prototypical GPCR, toward βarr recruitment, trafficking, and extracellular signal–regulated kinase 1 and 2 (ERK1/2) phosphorylation. We generate a set of systematically designed phosphorylation site mutants of the V_2_R and find that several phosphorylation sites can have distinct contribution in βarr interaction and functional responses. Some phosphorylation sites work concertedly to affect βarr recruitment, while others can have a decisive contribution on βarr recruitment, trafficking, and signaling even at individual levels. Molecular dynamics (MD) simulation provides structural insights into how specific phosphorylation sites on the receptor contribute toward the stability of βarr interaction and the interdomain rotation in βarrs upon activation. These findings help refine the conceptual framework of GPCR-βarr interaction and have direct implications for the paradigm of biased agonism.

## RESULTS

### Phosphorylation site mutants of human V_2_R

**Fig. 1:**
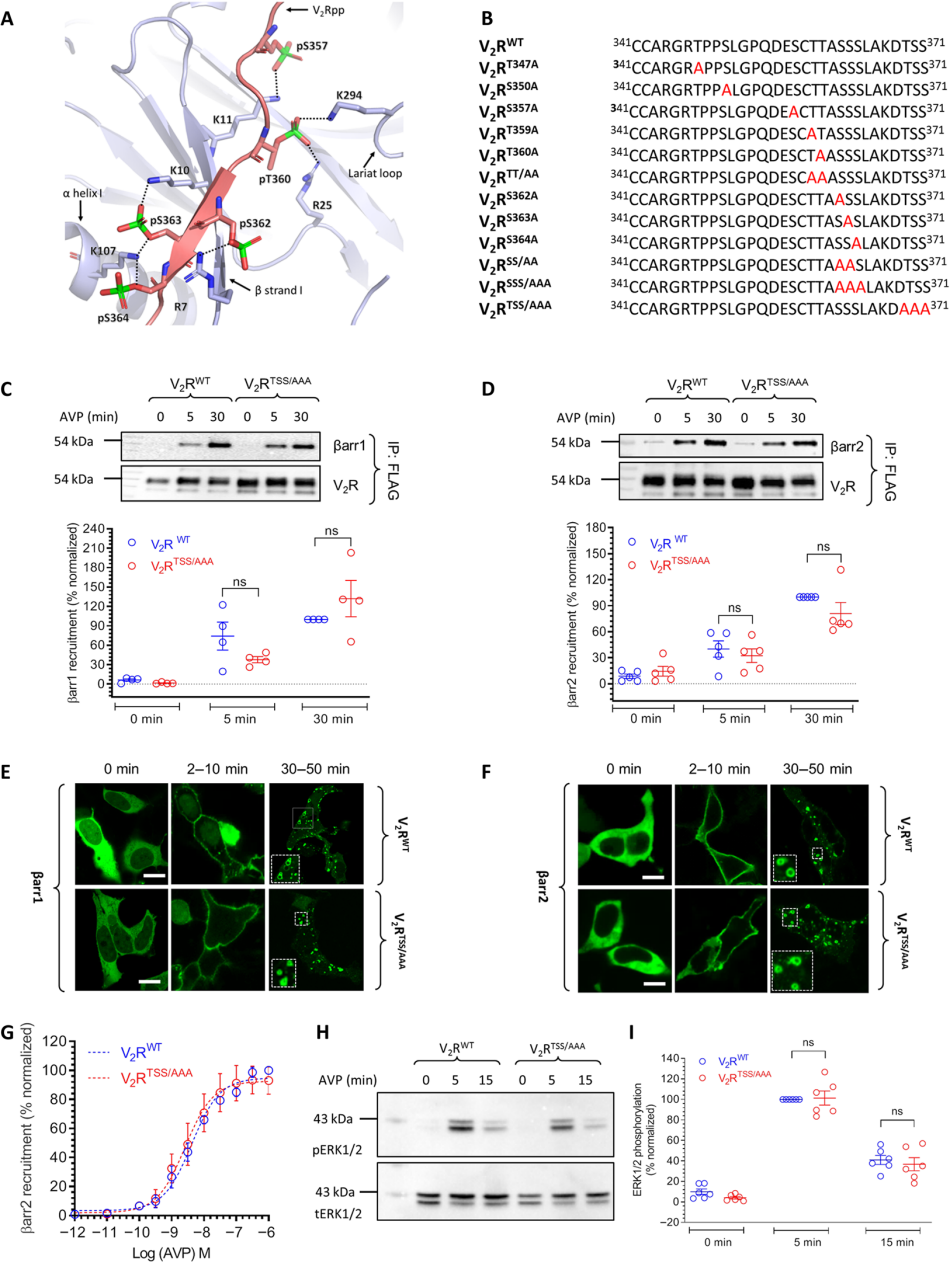
Phospho-site mutants of the V_2_R and contribution of the TSS cluster. (**A**) Structural snapshot of V_2_Rpp-βarr1 crystal structure depicting the interaction of phosphate groups with Lys/Arg (K/R) residues on βarr1, based on PDB ID: 4JQI. (**B**) C-terminal sequences of the V_2_R phosphorylation site mutants generated in this study. Mutated S/T residues are highlighted in red. (**C** and **D**) Mutation of TSS cluster does not significantly affect agonist-induced (100 nM AVP) βarr recruitment as assessed by co-IP experiment in HEK-293 cells. Representative images from four independent experiments (five for βarr2), and densitometry-based quantification of data (means ± SEM), normalized with respect to βarr co-IP for the V_2_R^WT^ at 30 min agonist stimulation (treated as 100%), are shown. (**E** and **F**) Agonist-induced trafficking of βarrs for the V_2_R^TSS/AAA^ mutant is similar to that of V_2_R^WT^ as assessed by confocal microscopy in HEK-293 cells expressing the receptor and βarr-mYFP. Cells were stimulated with 100 nM AVP, and representative images from three independent experiments at indicated time points are shown. Scale bars, 10 μm. (**G**) Agonist-induced recruitment of βarr2 for V_2_R^TSS/AAA^ mutant is also measured by Tango assay and found to be similar to that of V_2_R^WT^. Data (means ± SEM) from six independent experiments, each performed in duplicate and normalized with respect to the signal for V_2_R^WT^ at 1 μM AVP concentration (treated as 100%), are shown here. (**H** and **I**) Agonist-induced (100 nM AVP) ERK1/2 phosphorylation for V_2_R^TSS/AAA^ mutant is comparable to V_2_R^WT^ in HEK-293 cells at 5-min time point of agonist stimulation. A representative image from six independent experiments and densitometry-based quantification of the data, normalized with respect to the signal at 5 min for V_2_R^WT^ (treated as 100%). Data in (C), (D), and (I) are analyzed using two-way analysis of variance (ANOVA). ns, nonsignificant.

Previous studies have measured the role of V_2_R phosphorylation site clusters in βarr interaction and trafficking (*26*, *27*); however, the contribution of individual phosphorylation sites has not been explored. Therefore, we generated a series of V_2_R constructs with mutations of the potential phosphorylation sites either individually or in specific combinations, based on previously determined crystal structure of βarr1 in complex with V_2_R phosphopeptide (V_2_Rpp) (*21*) (Fig. 1, A and B). In addition to the eight phospho-sites present in V_2_Rpp, we also generated a mutant for the C-terminal Thr^369^/Ser^370^/Ser^371^ (V_2_R^TSS/AAA^) cluster that is not phosphorylated in V_2_Rpp (Fig. 1B). We measured the surface expression of each of these mutants in human embryonic kidney (HEK) 293 cells coexpressing either βarr1 or βarr2 using a previously described whole-cell enzyme-linked immunosorbent assay (ELISA) assay (*28*), and we observed that these mutants are expressed at comparable levels (fig. S1A). We then measured the interaction of V_2_R^TSS/AAA^ mutant with βarr1 and βarr2 using a cross-linking–based coimmunoprecipitation (co-IP) assay and observed that it interacts with βarrs at similar levels as the wild-type receptor (V_2_R^WT^) (Fig. 1, C and D). We further corroborated the similar pattern of βarr2 interaction of this mutant with V_2_R^WT^ using the Tango assay (Fig. 1G). We also evaluated the trafficking of βarrs upon stimulation of V_2_R^TSS/AAA^ mutant and observed a typical “class B pattern” similar to that of V_2_R^WT^ (Fig. 1, E and F, and fig. S2). Furthermore, agonist-induced ERK1/2 phosphorylation downstream of V_2_R^TSS/AAA^ was comparable to V_2_R^WT^ (Fig. 1, H and I). Together, these experiments suggest that the distal “TSS cluster” does not significantly contribute toward βarr recruitment, trafficking, and ERK1/2 phosphorylation.

### Contribution of Thr^347^, Ser^350^, and Ser^357^ in βarr recruitment and trafficking

**Fig. 2:**
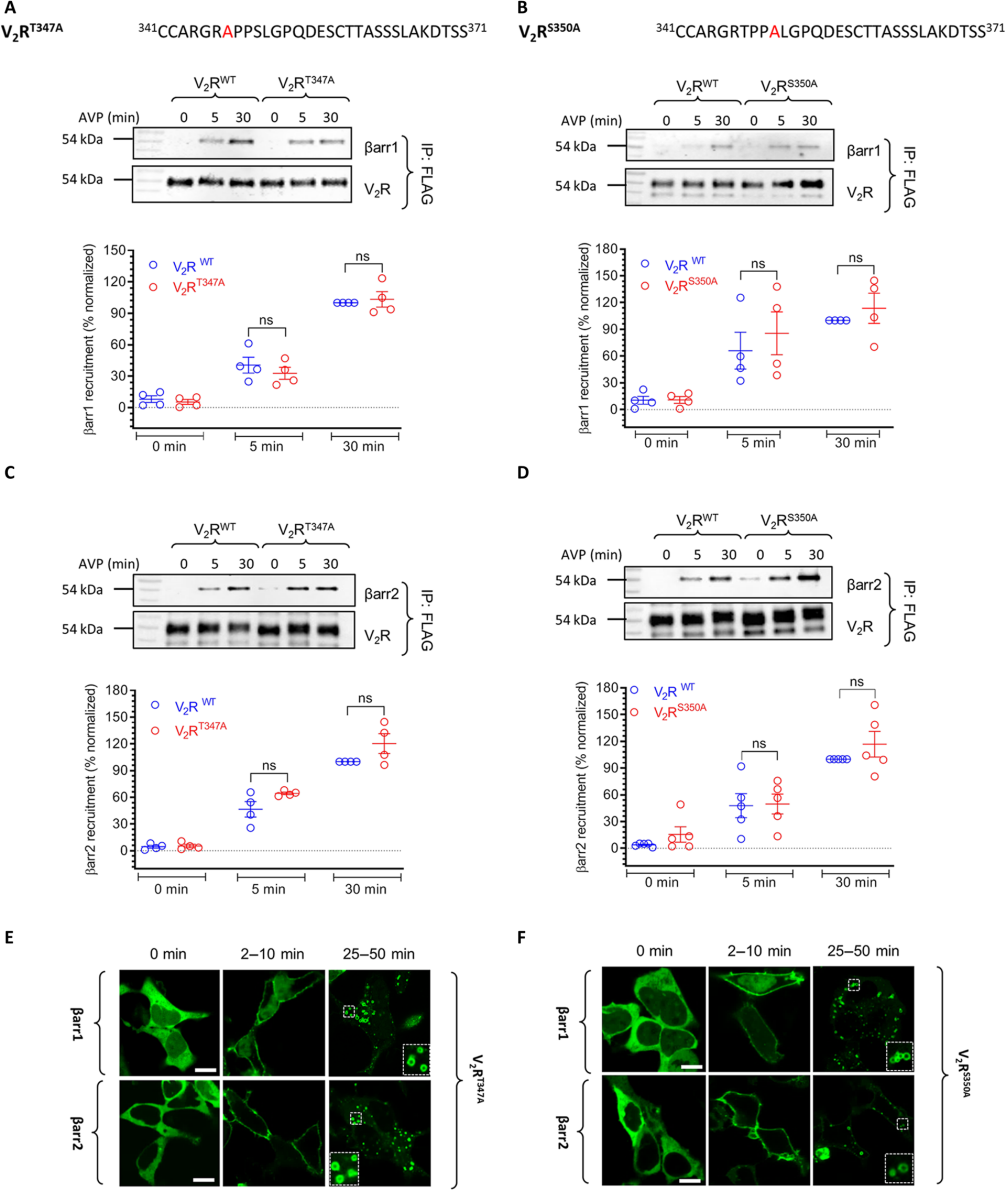
Mutations of T^347^ and S^350^ do not significantly affect βarr recruitment and trafficking. (**A** to **D**) Mutations of either T^347^ or S^350^ do not significantly affect agonist-induced (100 nM AVP) βarr recruitment as assessed by co-IP experiment in HEK-293 cells. Representative images from four independent experiments (five for S^350^ + βarr2), and densitometry-based quantification of data, normalized with respect to βarr co-IP for the V_2_R^WT^ at 30-min agonist stimulation (treated as 100%), are shown. Data are analyzed using two-way ANOVA. (**E** and **F**) Agonist-induced trafficking of βarrs for the V_2_R^T347A^ and V_2_R^S30A^ is similar to that of V_2_R^WT^ as assessed by confocal microscopy. HEK-293 cells expressing the indicated receptor mutant and βarr-mYFP were stimulated with 100 nM AVP, and representative images from three independent experiments at indicated time points are shown. Scale bars, 10 μm.

**Fig. 3:**
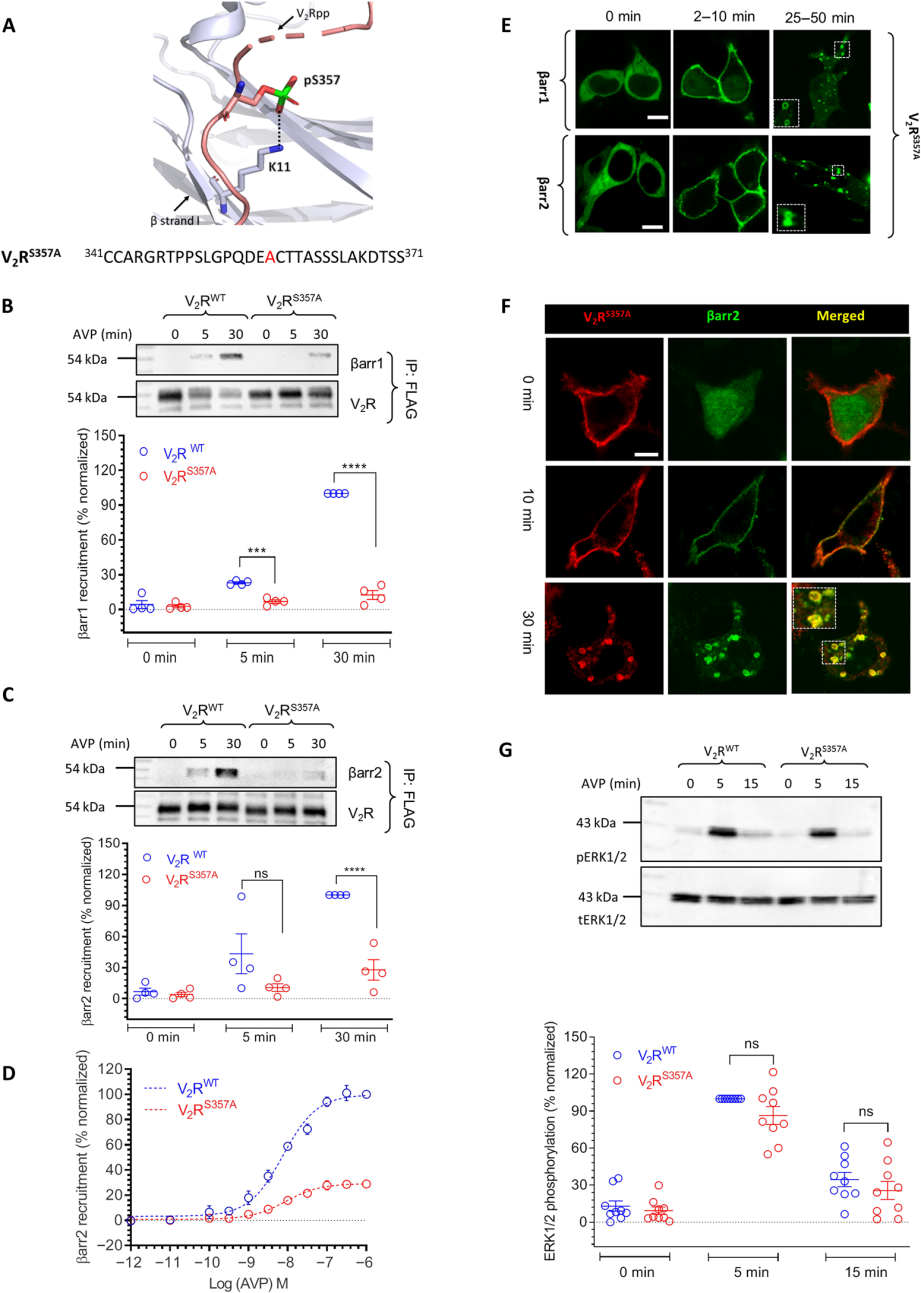
Mutation of S^357^ reduces βarr recruitment but does not affect trafficking patterns and ERK1/2 activation. (**A**) Structural snapshot of V_2_Rpp-βarr1 crystal structure depicting the interaction of the phosphate group at S^357^ with K^11^ in the β strand I of βarr1. (**B** and **C**) V_2_R^S357A^ mutant exhibits significant reduction in agonist-induced (100 nM AVP) βarr recruitment compared to V_2_R^WT^ as assessed by co-IP assay in HEK-293 cells. A representative image from four independent experiments and densitometry-based normalized data (means ± SEM) with respect to the signal for V_2_R^WT^ at 30 min (treated as 100%) is shown. Data are analyzed using two-way ANOVA (***P < 0.001 and ****P < 0.0001). (**D**) The reduction in agonist-induced βarr2 recruitment for V_2_R^S357A^ mutant compared to V_2_R^WT^ is further corroborated by Tango assay. Data (means ± SEM) from seven independent experiments, each performed in duplicate and normalized with respect to the signal for V_2_R^WT^ at 1 μM AVP concentration (treated as 100%), are shown here. (**E**) S^357^A mutation does not significantly alter the agonist-induced trafficking pattern of βarrs as measured qualitatively by confocal microscopy in HEK-293 cells expressing the receptor and βarr-mYFP. Cells were stimulated with 100 nM AVP, and representative images from three independent experiments at indicated time points are shown. Scale bars, 10 μm. (**F**) V_2_R^S357A^ exhibits agonist-induced (100 nM AVP) trafficking and colocalization with βarr2 in endosomal vesicles in HEK-293 cells. As visualized by confocal microscopy. Scale bar, 10 μm. (**G**) Agonist-induced (100 nM AVP) ERK1/2 phosphorylation downstream of V_2_R^S357A^ is similar to that of V_2_R^WT^ as measured in HEK-293 cells at indicated time points. A representative image from nine independent experiments, and densitometry-based quantification of data (means ± SEM), normalized with respect to the signal at 5 min for V_2_R^WT^, is shown in the bottom panel. Data are analyzed using two-way ANOVA.

In addition to phospho-site clusters, i.e., TT cluster (Thr^359^Thr^360^), SSS cluster (Ser^362^Ser^363^Ser^364^), and TSS cluster (Thr^369^Ser^370^Ser^371^), there are three scattered phosphorylation sites present in the C terminus of the V_2_R, which were also phosphorylated in V_2_Rpp, i.e., Thr^347^, Ser^350^, and Ser^357^. Of these, only Ser^357^ interacts with Lys^11^ on β strand I of βarr1 in the crystal structure of V_2_Rpp-βarr1 complex (Figs. 1A and 3A). We generated phospho-site mutants of V_2_R corresponding to each of these sites, i.e., Thr^347^, Ser^350^, and Ser^357^, and measured the interaction and trafficking of βarrs. We observed that V_2_R^T347A^ and V_2_R^S350A^ interacted efficiently with βarr1 and βarr2, similar to V_2_R^WT^ (Fig. 2, A to D). Moreover, the overall trafficking pattern of βarrs for the V_2_R^T347A^ and V_2_R^S350A^ was similar to that of V_2_R^WT^ (Fig. 2, E and F, and fig. S2). However, V_2_R^S357A^ exhibits a significant attenuation of βarr interaction compared to V_2_R^WT^ as measured by co-IP assay (Fig. 3, B and C). We further confirmed the interaction pattern of V_2_R^S357A^ with βarr2 using Tango assay and observed a significant reduction compared to V_2_R^WT^ (Fig. 3D), similar to that observed by co-IP (Fig. 3, B and C).

We next measured agonist-induced trafficking of βarrs for V_2_R^S357A^ using confocal microscopy. While the trafficking patterns of βarrs were qualitatively similar to V_2_R^WT^, i.e., surface translocation followed by robust internalization (Fig. 3E), we observed a reduced level of βarr trafficking to internalized vesicles for V_2_R^S357A^ compared to V_2_R^WT^ (fig. S2). To exclude the possibility of βarr internalization independent of the receptor (i.e., after dissociation from the receptor), as observed for a couple of different GPCRs previously (*29*, *30*), we also measured the colocalization of V_2_R^S357A^ with βarr2 in internalized vesicles. As presented in Fig. 3F, V_2_R^S357A^ was colocalized with βarr2 in internalized vesicles, suggesting that despite a reduced level of overall recruitment, the trafficking pattern of the receptor is not substantially altered. On the basis of the reduced level of βarr interaction, we anticipated a decrease in agonist-induced ERK1/2 phosphorylation for V_2_R^S357A^. Unexpectedly, we did not observe a significant difference compared to V_2_R^WT^, although a slight reduction in some experimental replicates was noticeable (Fig. 3G). Together, these data suggest that Thr^347^ and Ser^350^ are dispensable for βarr recruitment, at least in HEK-293 cells, but Ser^357^ plays an important role in βarr recruitment and trafficking without affecting ERK1/2 phosphorylation.

### Ser^362^ and Ser^363^ of SSS cluster are critical for βarr recruitment, trafficking, and ERK1/2 activation

**Fig. 4:**
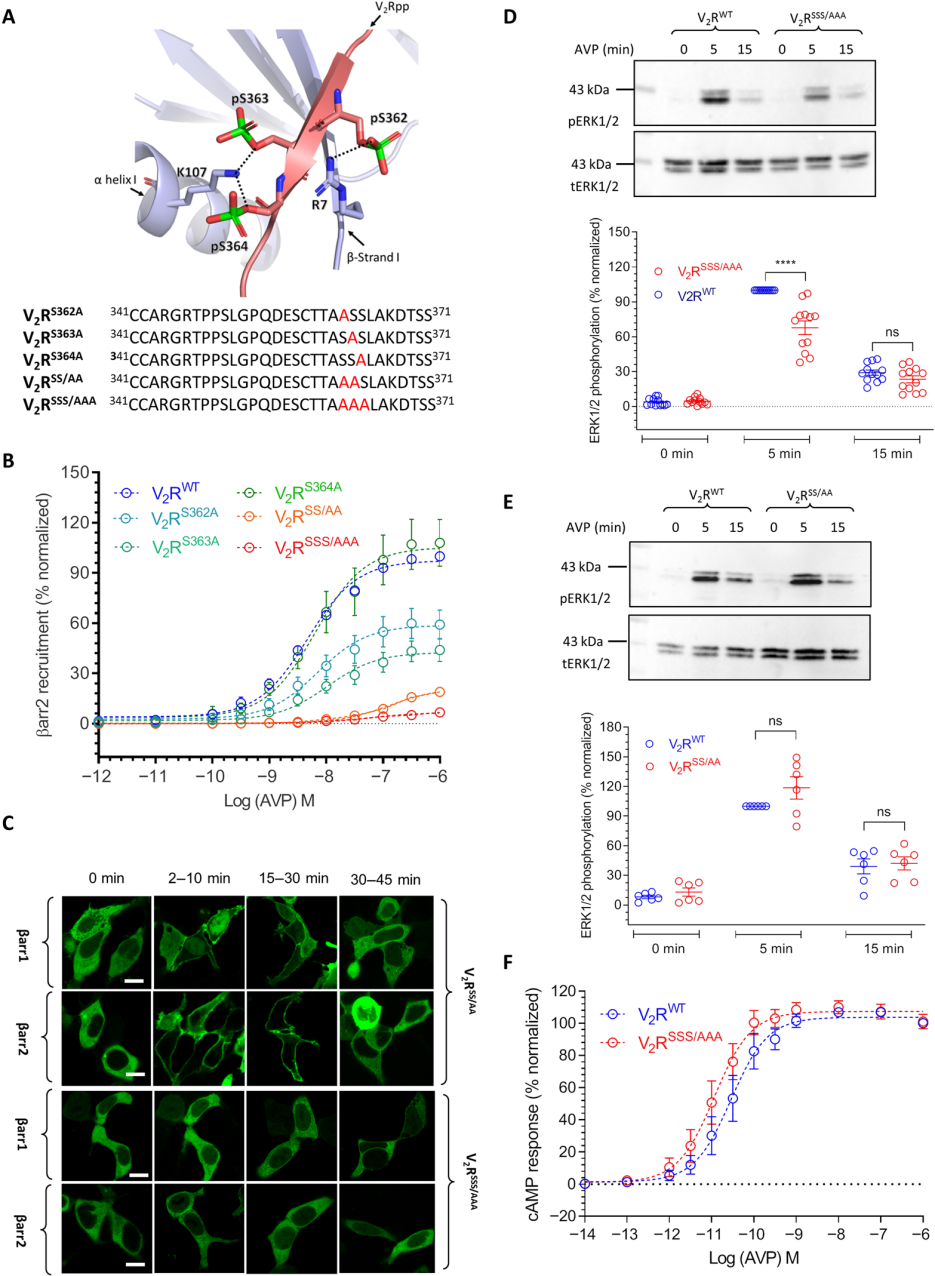
Concerted action of the phosphorylation sites in SSS cluster toward βarr recruitment, trafficking, and ERK1/2 activation. (**A**) Structural snapshot of V_2_Rpp-βarr1 crystal structure depicting the interaction of receptor-bound phosphate groups with K^107^/R^7^ in βarr1. The bottom panel shows the C-terminal sequences of V_2_R mutants with mutated S/T residues highlighted in red. (**B**) Tango assay reveals a prominent contribution of S^362^ and S^363^, but not of S^364^, in βarr2 recruitment. Simultaneous mutation of S^362^/S^363^ results in near-complete loss of βarr2 recruitment, which is reduced even further for the S^362^/S^363^/S^364^ (SSS/AAA) mutation. Data represent means ± SEM of eight independent experiments (six for V_2_R^SS/AA^), each carried out in duplicate and normalized with respect to the response at 1 μM concentration of AVP for V_2_R^WT^. (**C**) V_2_R^SS/AA^ mutant exhibits class A pattern of βarr translocation, and V_2_R^SSS/AAA^ displays no detectable translocation of βarr. Representative images from three independent experiments on HEK-293 cells expressing the indicated receptor mutants and βarr-mYFP, stimulated with 100 nM AVP, are shown. Scale bars, 10 μm. (**D** and **E**) Agonist-induced (100 nM AVP) ERK1/2 activation downstream of V_2_R^SS/AA^ is similar to that of V_2_R^WT^ but V_2_R^SSS/AAA^ exhibits a significant reduction at 5-min time point. Representative images from 12 (V_2_R^SSS/AAA^) and 6 (V_2_R^SS/AA^) independent experiments, and densitometry-based quantification of data (mean ± SEM), normalized with respect to the signal at 5-min time point for V_2_R^WT^ (treated as 100%), are shown. Data are analyzed using two-way ANOVA (****P < 0.0001). (**F**) Agonist-induced cAMP response for V_2_R^SSS/AAA^ is similar to that of V_2_R^WT^ as measured in HEK-293 cells using the GloSensor assay. Data (means ± SEM) from six independent experiments, each performed in duplicate, and normalized with respect to the response at 1 μM concentration of AVP for V_2_R^WT^ (treated as 100%). Two-way ANOVA suggests that the apparent difference in the cAMP dose–response curves for V_2_R^WT^ and V_2_R^SSS/AAA^ is not statistically significant.

Although previous studies have suggested a critical role of SSS cluster in V_2_R-βarr interaction and functional outcomes (*26*, *27*), a systematic analysis of the contribution of each of these phospho-sites individually has not been reported. Therefore, we generated five different constructs with mutations at either individual phospho-sites or in combination (Fig. 4A). While Ser^362^ interacts with Arg^7^ on β strand I in βarr1, Ser^363^ and Ser^364^ both are in direct contact with Lys^107^ on α helix I (Fig. 4A). We observed that Ser^362^ and Ser^363^ are important for βarr recruitment, while Ser^364^ does not seem to have a major role, when tested individually either by co-IP (fig. S3) or Tango assay (Fig. 4B). The double mutant, i.e., V_2_R^S362A/S363A^ (V_2_R^SS/AA^), is affected more markedly with respect to βarr recruitment compared to individual mutations (Fig. 4B and fig. S5A), while the triple mutant, i.e., V_2_R^S362A/S363A/364A^ (V_2_R^SSS/AAA^), is completely deficient in βarr recruitment (Fig. 4B and fig. S5B). We also observed that each of the individual phospho-site mutants exhibited typical “class B” pattern of βarr trafficking (fig. S4), similar to V_2_R^WT^. Quantification of confocal images, however, suggests a noticeable decrease in βarr localization, particularly βarr2, to internalized vesicles for V_2_R^S362A^ and V_2_R^S363A^ (fig. S2). The double mutant, i.e., V_2_R^SS/AA^ displays a “class A” pattern of βarr recruitment reflected by translocation of βarrs to the surface at early time points followed by redistribution in the cytoplasm (Fig. 4C). The triple mutant, i.e., V_2_R^SSS/AAA^, failed to exhibit any detectable translocation of βarrs (Fig. 4C), which also agrees with the lack of interaction observed in co-IP and Tango assays. We also measured agonist-induced ERK1/2 MAP kinase phosphorylation upon agonist stimulation of the double (V_2_R^SS/AA^) and the triple (V_2_R^SSS/AAA^) mutants and observed a significant reduction in V_2_R^SSS/AAA^-mediated ERK1/2 phosphorylation compared to V_2_R^WT^ at 5-min time point (Fig. 4D). There was no significant change in ERK1/2 phosphorylation mediated by the double mutant (V_2_R^SS/AA^) (Fig. 4E). Together, these data suggest that Ser^362^ and Ser^363^ contribute toward βarr interaction, and their collective contribution is more pronounced than individual sites. Furthermore, while Ser^364^ appears to be less important when tested individually, in the context of the triple mutant (V_2_R^SSS/AAA^), it seems to act concertedly with the other sites toward overall βarr recruitment, trafficking, and ERK1/2 activation.

### SSS^362/363/364^AAA mutant yields a G-protein–biased receptor

As the V_2_R^SSS/AAA^ mutant exhibits near-complete loss of βarr recruitment, it may potentially behave as a G-protein–biased mutant, if it maintains efficient G-protein coupling. To test this hypothesis, we measured agonist-induced cyclic adenosine 3′,5′-monophosphate (cAMP) response for this mutant and observed that it indeed exhibited a robust cAMP response, similar to V_2_R^WT^ (Fig. 4F). At a low agonist dose, this mutant is even more efficient in producing cAMP response compared to V_2_R^WT^, and the cAMP response appears to be more sustained, as expected, due to lack of βarr-mediated desensitization (fig. S7A). Therefore, V_2_R^SSS/AAA^ represents a βarr coupling–deficient, Gαs-biased V_2_R mutant that can be used in the future to delineate the specific contributions of G-protein and βarrs downstream of V_2_R.

### Thr^360^, but not Thr^359^, is critical for overall βarr recruitment, trafficking, and ERK1/2 phosphorylation

**Fig. 5:**
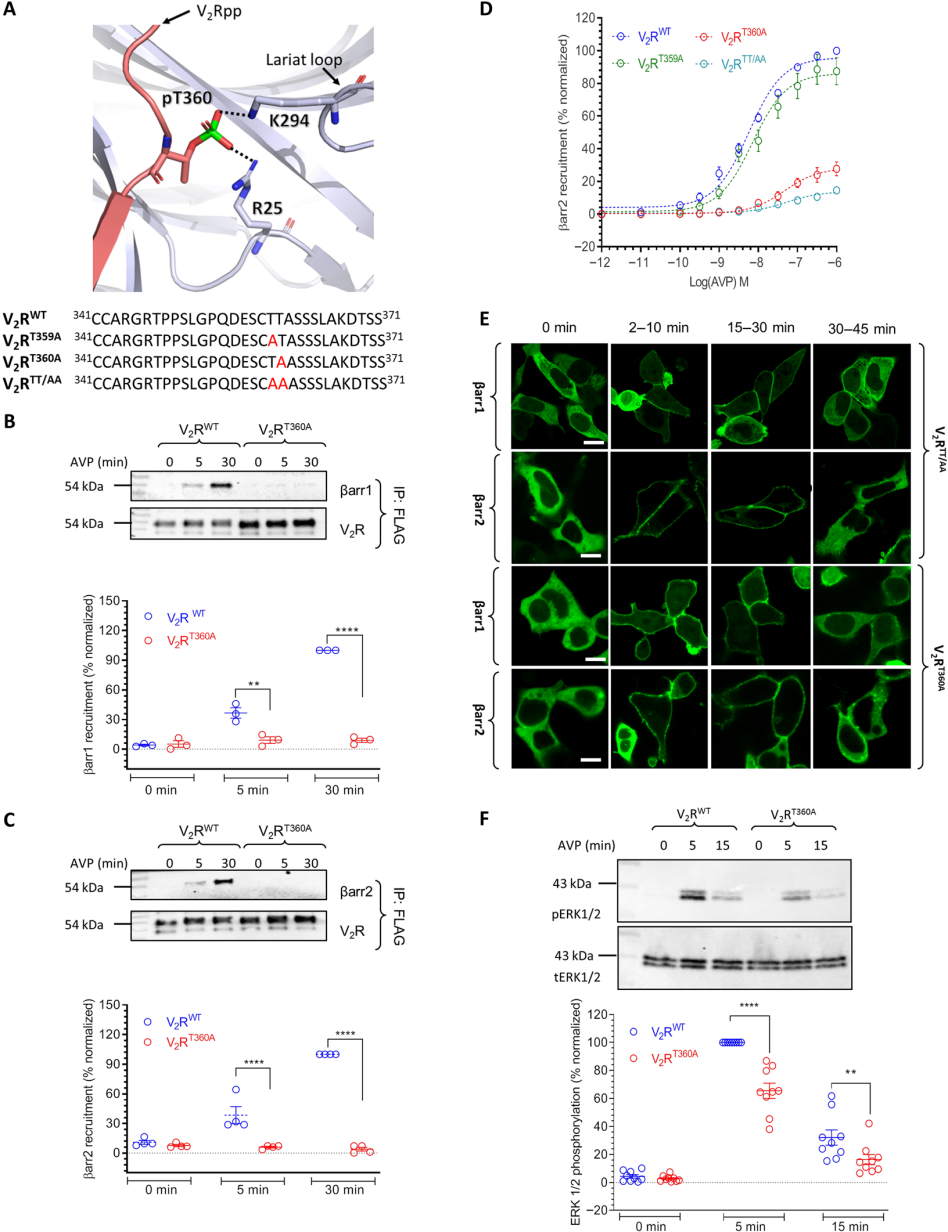
T^360^ plays a decisive role in βarr recruitment, trafficking, and ERK1/2 activation. (**A**) Structural snapshot of V_2_Rpp-βarr1 crystal structure depicting the interaction of T^360^ with K^294^ and R^25^ in βarr1. The bottom panel indicates C-terminal sequence of V_2_R phosphorylation site mutants with mutated S/T residues highlighted in red. (**B** and **C**) V_2_R^T360A^ mutant exhibits near-complete loss of βarr recruitment as measured in HEK-293 cells, stimulated with 100 nM AVP, using the co-IP assay. Representative images from three independent experiments (four for βarr2) and densitometry-based quantification of data (mean ± SEM), normalized with respect to the signal for V_2_R^WT^ at 30-min time point (treated as 100%), are shown. Data are analyzed using two-way ANOVA (**P < 0.01 and ****P < 0.0001). (**D**) Tango assay corroborates a major reduction in agonist-induced βarr2 recruitment for V_2_R^T360A^, which is reduced even further in the double phospho-site mutant, i.e., V_2_R^TT/AA^. Mutation of T^359^ alone does not lead to a significant reduction in βarr2 recruitment. Data (means ± SEM) from of seven independent experiments (eight for V_2_R^S360A^), normalized with respect to the response at 1 μM concentration of AVP for the V_2_R^WT^ (treated as 100%), are shown. (**E**) Mutation of T^360^ alone or in combination T^359^ (i.e., V_2_R^TT/AA^) confers a class A pattern of agonist-induced translocation of βarrs, and significant endosomal trafficking of βarrs is not observed even after prolonged agonist stimulation (100 nM AVP). Representative images from three independent experiments on HEK-293 cells expressing the indicated receptor mutants and βarr-mYFP, stimulated with 100 nM AVP, are shown. Scale bars, 10 μm. (**F**) V_2_R^T360A^ mutant displays a significantly reduced level of agonist-induced ERK1/2 phosphorylation compared to V_2_R^WT^ in HEK-293 cells stimulated with AVP (100 nM). A representative image from nine independent experiments and densitometry-based quantification of data (means ± SEM), normalized with respect to the signal at 5 min after agonist stimulation for V_2_R^WT^ (treated as 100%). Data are analyzed using two-way ANOVA (**P < 0.01 and ****P < 0.0001).

We next focused on the TT cluster and generated three different mutants as depicted in Fig. 5A. While Thr^359^ is not involved in any interaction with Lys/Arg in βarrs, Thr^360^ interacts with Arg^25^ in β strand II and Lys^294^ in the lariat loop (Fig. 5A). We observed that V_2_R^T359A^ exhibits efficient interaction with βarrs (fig. S6A); however, V_2_R^T360A^ displays significantly reduced interaction with βarrs (Fig. 5, B to D). The combination of these two phospho-sites, i.e., V_2_R^T359A/T360A^ (V_2_R^TT/AA^), exhibits even more pronounced loss of βarr interaction compared to V_2_R^T360A^ (Fig. 5D and fig. S6B). Notably, we also observed that V_2_R^T360A^ exhibits a typical class A pattern in terms of βarr trafficking as reflected by the surface translocation of βarrs followed by its redistribution in the cytoplasm (Fig. 5E). The double mutant V_2_R^TT/AA^ exhibited a pattern similar to V_2_R^T360A^ (Fig. 5E). On the other hand, V_2_R^T359A^ displayed a typical class B pattern of βarr translocation upon agonist stimulation (fig. S6C), although there appears to be a noticeable increase in the localization of βarr2 in internalized vesicles, compared to V_2_R^WT^ during the early time frame (fig. S2). We also measured agonist-induced ERK1/2 phosphorylation by V_2_R^T360A^ and V_2_R^TT/AA^ and observed a significant reduction compared to V_2_R^WT^ for both of these mutants (Fig. 5F and fig. S6D). Together, these data suggest that Thr^360^ plays a critical role in driving the interaction of V_2_R with βarrs as well as in determining the class B pattern of βarr trafficking and ERK1/2 phosphorylation, while Thr^359^ appears to be less important, at least in HEK-293 cells. Moreover, V_2_R^T360A^ also maintains an efficient G-protein coupling profile, as measured using cAMP responses via the GloSensor assay (fig. S7, B and C), and thus represents another G-protein–biased V_2_R mutant, similar to V_2_R^SSS/AAA^.

### Structural insights into receptor-βarr interaction and conformation

To gain structural and mechanistic insights into our findings, we used MD simulation using the V_2_Rpp-βarr1 crystal structure as a template (*21*). We first carried out classical unbiased simulation to monitor the dynamics of V_2_Rpp in the context of phospho-site mutations. Here, a quantitative measure of V_2_Rpp dynamics is obtained by computing the root mean square fluctuation (RMSF) per residue. We observed that the WT and mutated phosphopeptides corresponding to the mutants described above exhibited an overall similar RMSF profile (fig. S8). Expectedly, we observed higher RMSF at the N-terminal (346 to 348) and the C-terminal ends (366 to 372) of the phosphopeptide, while two stretches in the middle that adopt an extended β strand and pack against the β strand I of βarr1 via backbone interactions displayed much lower RMSF profile (fig. S8).

**Fig. 6:**
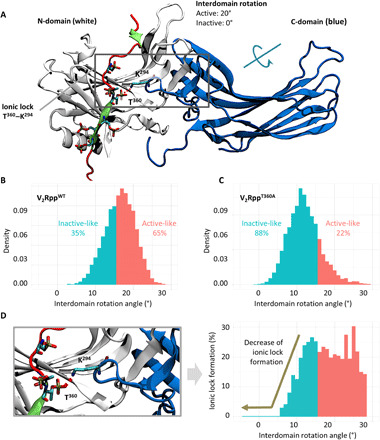
MD simulation yields structural insights into βarr recruitment and conformation. (**A**) Structural snapshot of the V_2_Rpp-βarr1 complex depicting the ionic lock between T^360^ in V_2_Rpp and K^294^ in βarr1. (**B**) Distribution of the interdomain rotation angles adopted by βarr1 in complex with the V_2_Rpp as measured by MD simulation. We observed a peak with an interdomain rotation of about 17° which agrees well with the experimental data and previous simulation studies. (**C**) Distribution of the interdomain rotation angles adopted by βarr1 upon V_2_R^T360A^ mutation where the peak is shifted to about 11°. For V_2_R^T360A^ mutation, the fraction of active conformers with larger interdomain rotation is also reduced compared to V_2_Rpp. (**D**) The stability of the ionic lock between T^360^ and K^294^ as a function of the interdomain rotation angle reveals that the ionic lock formation reduces with the decrease in the interdomain rotation. βarr1 conformers with an interdomain rotation angle of less than 11° display a lower frequency of ionic lock formation. Note that the frequencies do not reach more than 30% due to a large flexibility of the lariat loop, which gives the ionic lock a rather transient character.

We found that Thr^360^ is repeatedly the most stable position in all simulated systems (fig. S8). This indicates that Thr^360^ is an anchor point for the binding of phosphorylated receptor tail to βarrs and provides a potential mechanistic basis for a marked reduction in βarr recruitment. Thr^360^ is a part of the extended β strand in the middle of V_2_Rpp, and it interacts with Lys^294^ in the lariat loop of βarr1 through a strong electrostatic interaction (Fig. 6A). Structurally, Thr^360^ is at the center of a three-way connection between the N-domain, the V_2_Rpp, and the C-domain of βarr1 through the Thr^360^-Lys^294^ ionic lock (Fig. 6A). Thus, it is tempting to speculate that the Thr^360^-Lys^294^ ionic lock may be a crucial determinant for the interdomain rotation between the N- and C-domain observed upon V_2_Rpp binding and activation of βarr1.

To test this possibility, we first assessed the interdomain rotation angle of the βarr1 in complex with the V_2_Rpp and observed an average rotation angle of 17°, which agrees well with experimental observation (*21*) and previous simulation experiments (*31*) (Fig. 6B). The average interdomain rotation angle changed to about 11° for V_2_Rpp^T360A^ (Fig. 6C). In complex with V_2_Rpp, βarr1 is able to sample a broad spectrum of conformations during activation where larger interdomain rotation occurs at high probability, while the smaller interdomain rotation has relatively lower probability. However, in the context of V_2_Rpp^T360A^, conformations with smaller interdomain rotation become markedly more populated (Fig. 6C). This marked alteration is quantitatively visible upon comparison of active-like populations (i.e., with an interdomain rotation angle >15°) between V_2_Rpp and V_2_Rpp^T360A^ analysis (Fig. 6, B and C).

We further computed the stability of the ionic lock (Thr^360^-Lys^294^) across all sampled activation states of the βarr1-V_2_Rpp complex (Fig. 6D). We observed that the ionic lock stability directly correlates with the interdomain rotation angles (Fig. 6D). There is a marked reduction in the ionic lock formation in inactive-like βarr1 conformations with interdomain rotation angles <15°. This is in agreement with the difference in average interdomain rotation angles and conformational distribution between V_2_Rpp (17°; ionic lock present) and V_2_Rpp^T360A^ (11°; ionic lock absent) as mentioned above (Fig. 6, B and C). Together, these simulation data underscore the role of Thr^360^-Lys^294^ ionic lock as an important element in stabilizing the relative orientation of the N- and the C-domain in βarr1 upon activation, which may, in turn, fine-tune the functional responses.

## DISCUSSION

GPCR phosphorylation is a key determinant of βarr interaction and imparting specific conformational signatures linked to distinct functional responses (*19*, *32*). Previous studies have proposed a direct link between the receptor phosphorylation patterns and ensuing functional outcomes; however, integrating these findings in a structural framework still remains somewhat preliminary. Here, we find that even a single phosphorylation site in V_2_R, i.e., Thr^360^ can have a decisive contribution in βarr recruitment by serving as an anchor point for stable interaction. Moreover, it can also critically influence the activation-dependent conformational changes such as interdomain rotation angle via the formation of an important ionic lock with Lys^294^ in βarr1. This observation underscores the importance of spatial positioning of key phosphorylation sites in the receptor as crucial parameter, in addition to previously determined phospho-clusters (*27*) and phospho-codes (*18*). The importance of spatial localization of the key phospho-sites is further corroborated by the observation that Thr^359^ positioned right next to Thr^360^ has no measurable effect on βarr recruitment or trafficking. The phosphate group on Thr^359^ points away from the Lys^294^ in V_2_Rpp-βarr1 crystal structure, suggesting that its spatial positioning is unsuitable for interacting with the lariat loop, even in the context of Thr^360^ phospho-site mutation. As Lys^294^ is conserved in βarrs, it is possible that its interaction with suitably positioned receptor phosphates may contribute generally toward βarr activation, conformational change, and functional responses, although its mutation does not appear to significantly affect the overall interaction between the selected GPCRs and βarrs as reported in a recent study (*33*). Future studies designed to probe this in detail with a set of different GPCRs may shed further light on this interesting conjecture.

Although previous studies have reported a collective role of triple serine cluster, i.e., Ser^362/363/364^ in βarr trafficking (*26*, *27*, *34*), our study reveals a concerted contribution of individual phospho-sites present in this cluster. While individual mutation of Ser^362^ and Ser^363^ significantly reduces βarr binding but not the trafficking pattern, Ser^364^ is mostly dispensable. However, a combination of Ser^362^ and Ser^363^ diminishes βarr recruitment further and also changes the trafficking pattern from class B to class A. Although Ser^364^ by itself does not appear to have a major role, in conjunction with Ser^362/^Ser^363^ mutation, it facilitates complete abrogation of βarr recruitment. This observation implies that contribution of some phospho-sites present in a cluster may be evident only upon a combinatorial analysis. Moreover, as the G-protein coupling of the V_2_R^SSS/AAA^ mutant remains primarily unaltered, it essentially imparts G-protein bias on V_2_R. Thus, it may serve as a promising tool to further investigate structural and functional aspects of V_2_R-effector coupling and signaling responses (*35*). An intriguing pattern that emerges from our study is that the extent of βarr recruitment and ERK1/2 phosphorylation do not necessarily correlate with each other. For example, V_2_R^S357A^ and V_2_R^SS/AA^ mutants have significantly reduced levels of βarr recruitment; however, their agonist-induced ERK1/2 phosphorylation patterns are mostly similar to V_2_R^WT^. While the contribution of both G-proteins and βarrs in ERK1/2 activation downstream of GPCRs is well established, our data suggest that even a transient interaction or an overall lesser extent of βarr interaction is sufficient to drive robust ERK1/2 activation. This notion is also confirmed by previous studies on the β1 adrenergic receptor system (*29*, *30*).

A recent study using the rhodopsin-visual-arrestin system has proposed that phospho-sites can be categorized as the key sites, modulatory sites, and inhibitory sites and hypothesize that a similar pattern may exist for other GPCRs as well (*36*). While we do observe that Ser^357^ and Thr^360^ mutation significantly decreases βarr recruitment, we did not find an inhibitory role of any of the phospho-sites in the V_2_R. Nonetheless, future studies with additional receptor systems may provide experimental evidence, or lack thereof, for this provocative hypothesis. Moreover, recent studies using intrabody sensors have suggested conformational diversity in GPCR-βarr complexes despite an overall similar recruitment profile and trafficking patterns (*37*–*39*). Therefore, it would be very interesting to analyze the conformational signatures of βarrs in complex with these V_2_R mutants in further studies. It is also worth noting that although we observe that the mutation of some putative phosphorylation sites do not have a significant effect on βarr recruitment and trafficking, we cannot discern whether these sites are phosphorylated, or not, in HEK-293 cells or if they are completely dispensable. This remains an open question for future investigation especially considering the emerging evidence for cell type– and tissue-specific GPCR phosphorylation and signaling mechanisms (*40*). Furthermore, a kinetic analysis of agonist-induced βarr recruitment for these receptor mutants may yield additional insights into the potential contribution of different phosphorylation sites in transient interactions between the receptor and βarrs. It is also worth noting that the crystal structure of βarr1 in complex with V_2_Rpp was determined using rat βarr1, while the constructs used here for co-IP experiments are of bovine origin. Although the sequences of βarr1 are highly similar across different species, βarr2 displays slightly higher sequence divergence, and a minor effect of such sequence differences on βarr conformation and functional outcomes cannot be completely ruled out.

In conclusion, we find that even single phosphorylation sites on GPCRs may encode critical determinants for βarr interaction and trafficking. Moreover, individual sites in a cluster may act in a concerted fashion to impart distinct βarr interaction and trafficking patterns. Our data also reveal that a single phospho-site may act as an anchor point for the stability of interaction and directing the degree of interdomain rotation during the activation process. This study provides a missing piece in the paradigm of GPCR-βarr interaction using V_2_R as a model system, and it also offers a framework that may potentially have general applicability for other GPCRs as well.

## MATERIALS AND METHODS

### General reagents, cell culture, and expression plasmids

Most of the general chemicals used here for molecular biology, biochemistry, and cell biology experiments were purchased from Sigma-Aldrich. Trypsin-EDTA, Hank’s balanced salt solution (HBSS), and penicillin-streptomycin solution were purchased from Thermo Fisher Scientific. The expression constructs for the wild-type human V_2_R , bovine βarr1, and βarr2 have been described previously (*39*), and rat βarr1/2-mYFP plasmids were obtained from Addgene (cat. nos. 36916 and 36917). The phosphorylation site mutants were generated using Q5 Site-Directed Mutagenesis Kit (NEB) and sequence-verified (Macrogen). V_2_R agonist AVP (arginine-vasopressin) was either purchased from Sigma-Aldrich or synthesized (GenScript). HEK-293 cells (American Type Culture Collection) were maintained and cultured in DMEM (Dulbecco’s modified Eagle’s medium) supplemented with 10% fetal bovine serum, penicillin (100 U/ml), and streptomycin (100 μg/ml). Cells were cultured in 10-cm dishes (Corning) at 37°C under 5% CO_2_ and passaged at 70 to 80% confluency using 0.05% trypsin-EDTA for detachment.

### DNA transfection and surface expression of V_2_R mutants

For various assays described in the manuscript, HEK-293 cells at 60 to 70% confluency were transfected with the indicated constructs using polyethylenimine (PEI) as the transfection reagent at a typical DNA:PEI ratio of 1:3. Surface expression of V_2_R constructs was measured using whole-cell surface ELISA as described previously (*28*). Briefly, 24 hours after transfection, 0.2 million transfected cells were seeded into each well of 24-well plates, precoated with 0.01% poly-d-lysine. After another 24 hours, cells were fixed with 4% (w/v) paraformaldehyde (pH 6.9) on ice for 20 min and washed three times with 1× tris-buffered saline (TBS) buffer [150 mM NaCl and 50 mM tris-HCl (pH 7.4)]. Subsequently, nonspecific sites were blocked with 1% bovine serum albumin (BSA; prepared in 1× TBS) for 90 min, followed by the incubation of cells with horseradish peroxidase (HRP)–coupled anti-Flag M2 antibody (Sigma-Aldrich; cat. no. A8592) at a dilution of 1:10,000, prepared in 1% BSA for 90 min. Cells were then washed three times with 1× TBS, and 200 μl of tetramethylbenzidine (TMB) ELISA substrate (GenScript) was added to each well. Once the blue color appeared in the wells, the reaction was stopped by transferring 100 μl of the solution to a different 96-well plate already containing 100 μl of 1 M H_2_SO_4_. Absorbance was measured at 450 nm in a multimode plate reader (PerkinElmer, Victor X4). For normalization of signal across different wells, cell density was estimated using Janus Green staining. TMB solution was removed from the wells; cells were washed three times with 1× TBS followed by incubation with 0.2% (w/v) Janus Green for 20 min. Afterward, cells were washed three times with distilled water, 800 μl of 0.5 N HCl was added to each well, and 200 μl of this solution was used for measuring the absorbance at 595 nm. Normalized surface expression of V_2_R constructs was calculated as the ratio of absorbance at 450 and 595 nm.

### Chemical cross-linking and co-IP

For measuring agonist-induced V_2_R-βarr interaction, HEK-293 cells expressing the corresponding proteins were starved using incomplete DMEM for 6 hours, followed by stimulation with AVP (100 nM) for indicated time points. Afterward, cells were collected, lysed by douncing in lysis buffer [20 mM Hepes (pH 7.4), 150 mM NaCl, 1 mM phenylmethylsulfonyl fluoride, 2 mM benzamidine, and 1× PhosStop], followed by the addition of freshly prepared 1 mM DSP (dithiobis succinimidyl-propionate) (Sigma-Aldrich; cat. no. D3669). After 40 min of DSP cross-linking with continuous tumbling, the reaction was quenched with 100 mM tris-HCl (pH 8.5), and then cellular lysate was solubilized with 1% (v/v) MNG (maltose neopentyl glycol) for 1 hour at room temperature. Subsequently, the solubilized proteins were separated by centrifugation at 15,000 rpm for 30 min, and pre-equilibrated anti-Flag M1 antibody sepharose beads were added. Samples were supplemented with 2 mM CaCl_2_, and bead binding was allowed to occur for 2 hours at 4°C with gentle tumbling. The beads were washed three times each with low-salt buffer [20 mM Hepes (pH 7.4), 150 mM NaCl, 2 mM CaCl_2_, and 0.01% (v/v) MNG] and high-salt buffer [20 mM Hepes (pH 7.4), 350 mM NaCl, 2 mM CaCl_2_, and 0.01% (v/v) MNG], alternatively, to remove unbound and nonspecifically bound proteins. Last, the bound proteins were eluted using elution buffer [20 mM Hepes (pH 7.4), 150 mM NaCl, 2 mM EDTA, 0.01% MNG, and Flag peptide (250 μg/ml)]. A similar protocol was followed for the control co-IP experiment (presented in fig. S1B), except that anti-HA antibody agarose beads were used, instead of M1 antibody agarose. Receptor and βarrs in co-IP samples were detected by Western blotting by first using rabbit anti-βarr antibodies (1:5000; CST, cat. no. 4674), followed by reprobing the blots with HRP-conjugated anti-Flag M2 antibody (1:5000; Sigma-Aldrich, cat. no. A8592). Protein bands on the Western blots were visualized using a ChemiDoc imaging system (Bio-Rad). For densitometry-based quantification of co-IP samples, the band intensities on the Western blots were measured using either the Image Lab software (Bio-Rad), or ImageJ, and plotted in GraphPad Prism. The anti-Flag M2 antibody blots detecting the immunoprecipitation of various V_2_R constructs typically exhibited two bands, and both bands were used for densitometry. These two bands presumably indicate mature (fully glycosylated) and immature (partially glycosylated) receptor populations.

### GloSensor assay for measuring agonist-induced cAMP response

For measuring cAMP response for V_2_R constructs, HEK-293 cells were cotransfected with the indicated receptor construct and 22F plasmid (Promega). Twenty-four hours after transfection, cells were detached from the plates, centrifuged, and resuspended in buffer [1× HBSS supplemented with 20 mM Hepes (pH 7.4)] containing luciferin (0.5 mg/ml; GoldBio). Cells were seeded in white, glass-bottom 96-well plates at a density of 80,000 to 100,000 cells per well in 100 μl volume per well. Afterward, the 96-well plate was kept at 37°C for 1.5 hours under 5% CO_2_, followed by an additional incubation at room temperature for 30 min. Subsequently, the basal luminescence readings were recorded using a plate reader (Victor X4, PerkinElmer), followed by the addition of indicated concentrations of agonist (AVP) and recording of luminescence for up to 1 hour. Data were corrected for baseline signal and normalized with respect to highest concentration (1 μM) of AVP and plotted in GraphPad Prism. The GloSensor experiments were performed at an endogenous level of βarrs, i.e., without βarr overexpression, and only the indicated receptor constructs together with 22F plasmid were transfected for overexpression.

### Agonist-induced ERK1/2 phosphorylation

Agonist-induced ERK1/2 phosphorylation was measured as a readout of βarr signaling downstream of V_2_R mutants following the previously described protocol (*41*). Briefly, HEK-293 cells were transfected with 0.5 μg of indicated V_2_R constructs, and 24 hours after transfection, cells were seeded into six-well plates at a density of about 1 million cells per well. The next day, cells were serum-starved in DMEM for 6 hours followed by stimulation with 100 nM AVP for indicated time points, culture medium was aspirated, and cells were lysed in 100 μl of 2× SDS gel loading buffer. Cellular lysates were heated at 95°C for 15 min, followed by centrifugation at 15,000 rpm for 10 min, and 10 μl of samples was used for SDS–polyacrylamide gel electrophoresis. Phosphorylated ERK1/2 signal was detected by Western blotting using anti–phospho-ERK1/2 antibody (1:5000; CST, cat. no. 9101) followed by reprobing of the blots with anti–total-ERK1/2 antibody (1:5000; CST, cat. no. 9102). Signal on the Western blots was detected using the ChemiDoc imaging system (Bio-Rad), and densitometry-based quantification was carried out using Image Lab software or ImageJ. ERK1/2 phosphorylation experiments were performed at an endogenous level of βarrs, i.e., without βarr overexpression, and only the indicated receptor constructs were transfected for overexpression.

### Confocal microscopy

To visualize the agonist-induced trafficking of βarrs upon stimulation of V_2_R mutants, HEK-293 cells were cotransfected with the indicated V_2_R construct and βarr1/2-mYFP. Twenty-four hours after transfection, 1 million cells were seeded in glass bottom confocal imaging plates, precoated with 0.01% poly-d-lysine. After another 24 hours, cells were serum-starved for 2 to 3 hours and then subjected to live cell imaging using Carl Zeiss LSM780NLO confocal microscope fitted with 32× array GaAsP descanned detector (Zeiss) under 63×/1.40 numerical aperture objective with oil immersion. First, the cytoplasmic distribution of βarrs was recorded under basal conditions, followed by stimulation of cells and recording of βarrs localization in indicated time frame. For the two-color confocal imaging to measure the colocalization of the V_2_R^S357A^ and βarr2 (presented in Fig. 3F), transfected cells (24 hours after transfection) were seeded onto glass coverslips, precoated with 0.01% poly-d-lysine, and allowed to grow for another 24 hours. The next day, cells were serum-starved for 2 hours followed by stimulation with AVP (100 nM) for 0, 10, and 30 min. Subsequently, the cells were fixed with 4% paraformaldehyde prepared in 1× phosphate-buffered saline (PBS), permeabilized with 0.01% Triton X-100 for 10 min. For staining the receptor, cells were incubated with DyLight 594 conjugated anti-Flag M1 antibody (at 1:100 dilution prepared in 1% BSA solution) for 1 hour at room temperature. Afterward, cells were washed several times with 1× PBS, and then the coverslips containing fixed cells were mounted onto glass slides using VectaShield H-1000 mounting medium (VectaShield). The slides were air-dried for 20 to 30 min before imaging by confocal microscopy. Multiline argon laser source is used for green channel (mYFP), and for the red channel (DyLight 594), a diode pump solid state laser source was used. All the settings including laser intensity and pinhole settings were maintained in the same range for parallel set of experiments, and the filter excitation regions and bandwidths were adjusted for the channels to avoid any spectral overlap.

For the quantification of agonist-induced localization of βarrs for different V_2_R mutants, confocal images from multiple fields in at least three independent experiments were manually scored. Confocal images captured during 1 to 8 and 9 to 60 min after agonist stimulation were grouped under early and late time frames, respectively. The localization of βarrs was scored as surface and internalized on the basis of YFP fluorescence in the plasma membrane and punctate structures in the cytoplasm, respectively. In other words, cells with βarr-YFP in the plasma membrane are scored under “surface” category, while the cells displaying βarr-YFP in punctate structures in the cytoplasm are counted under “internalized” category. All images in the field were used for counting, and the data are plotted as percentage of βarr localization pattern from more than hundred cells for each condition. In a scenario where βarrs were present in both, the membrane and in punctate structures, cells with more than three punctae in the cytoplasm were scored under internalized category. To minimize any bias in scoring, the same set of images was analyzed by three different individuals and cross-checked. Data were plotted using GraphPad Prism software.

### Tango assay for βarr2 recruitment

Tango assay was used to measure agonist-used βarr2 recruitment following a previously described protocol (*42*). Briefly, HTLA cells expressing a tTA-dependent luciferase reporter and βarr2-TEV fusion protein were transfected with indicated V_2_R constructs. The V_2_R constructs for Tango assay compose of a receptor-coding region, followed by a TEV cleavage site and the tTA transcription factor coding sequence. Approximately 3 million HTLA cells were seeded onto a 10-cm cell culture plate, transfected with indicated receptor constructs, and 24 hours after transfection, cells were detached using trypsin-EDTA solution. Cells were resuspended in complete DMEM and seeded into 96-well white polystyrene plates at a density of about 50,000 cells per well. After another 24 hours, cells were stimulated with indicated concentrations of AVP for 7 to 8 hours. Subsequently, the growth medium was removed from the wells, and 100 μl of luciferin solution (0.5 mg/ml in 1× HBSS buffer) was added to each well. The luminescence signal was measured at 450 nm, and data were baseline-corrected, plotted, and analyzed using nonlinear regression in GraphPad Prism software.

### MD simulation

#### System setup and simulation

To generate all simulated complexes, we used the structure of V_2_Rpp in complex with βarr1 [Protein Data Bank (PDB) code: 4JQI]. The cocrystallized Fab30 antibody was removed, and missing fragments in the βarr1 and V2Rpp structures were modeled using the loop modeler module available in the MOE package (www.chemcomp.com). The complexes were solvated (TIP3P water) and set to an ionic strength of 0.15 M sodium chloride. Simulation parameters were obtained from the Charmm36M force field (*43*). In the simulation protocol, we adhere to the guidelines of the GPCRmd consortium (*44*). Systems generated this way were simulated using the ACEMD software (*45*). To allow rearrangement of waters and side chains, we carried out a 25-ns equilibration phase in NPT conditions with restraints applied to backbone atoms. The time step was set at 2 fs, and the pressure was kept constant, using the Berendsen barostat. After NPT equilibration, systems were subjected to production runs (NVT ensemble) for 1 μs in four parallel runs. Simulation runs of the V_2_R^WT^ and V_2_R^T360A^ systems were extended to 2 μs, amassing a total of 8 μs per system. For each NVT run, we used a 4-fs time step. In all runs, temperature was kept at 300 K using the Langevin thermostat, and hydrogen bonds were restrained using the RATTLE algorithm. Nonbonded interactions were cut off at 9 Å with a smooth switching function applied at 7.5 Å.

#### Analysis

To evaluate C-terminal tail stability, we aligned the system using backbone atoms of arrestin. Afterward, RMSF values were calculated for the Cα atoms of the C-terminal tail. The interdomain rotation angle was used as a metric to assess the activation state of βarr1. We computed the displacement of the C-domain relative to the N-domain between the inactive (PDB code: 1G4R) and active βarr1 crystal structures (PDB code: 4JQI) as previously described (*31*). The corresponding script was provided by N. Latorraca. Using obtained values of the rotational angles, we divided the simulation frames into groups with a bin width of 1. For each bin of rotation angle, we assessed the stability of the ionic lock between residue T360 of the peptide and K294 of the lariat loop. A salt bridge was defined as the distance between heavy polar atoms of those residues with less than 4 Å.

### Statistical analysis and data presentation

Experiments were repeated at least three times, and data were plotted and analyzed using GraphPad Prism software. The details of data normalization, statistical analysis, and *P* values are included in the corresponding figure legends.

## Supplementary Material

abb8368_SM.pdf

## SUPPLEMENTARY MATERIALS

Supplementary material for this article is available at http://advances.sciencemag.org/cgi/content/full/6/37/eabb8368/DC1

## Appendix

View/request a protocol for this paper from *Bio-protocol*.
