# Supplementary material for: Distinct phosphorylation sites in a prototypical GPCR differently orchestrate β-arrestin interaction, trafficking, and signaling
Source: Sci Adv. 2020 Sep 11;6(37):eabb8368. doi: 10.1126/sciadv.abb8368 (PMC7486103; doi:10.1126/sciadv.abb8368)
Supplement: abb8368_SM.pdf [file abb8368_SM.pdf]

Science Advances
AAAS

advances.sciencemag.org/cgi/content/full/6/37/eabb8368/DC1

Supplementary Materials for

Distinct phosphorylation sites in a prototypical GPCR differently orchestrate β-arrestin interaction, trafficking, and signaling

Hemlata Dwivedi-Agnihotri, Madhu Chaturvedi, Mithu Baidya, Tomasz Maciej Stepniewski, Shubhi Pandey, Jagannath Maharana, Ashish Srivastava, Natarin Caengprasath, Aylin C. Hanyaloglu, Jana Selent*, Arun K. Shukla*

*Corresponding author. Email: jana.selent@upf.edu (J.S.); arshukla@iitk.ac.in (A.K.S.)

Published 11 September 2020, *Sci. Adv.* **6**, eabb8368 (2020)
DOI: 10.1126/sciadv.abb8368

This PDF file includes:

Figs. S1 to S8

Figure S1

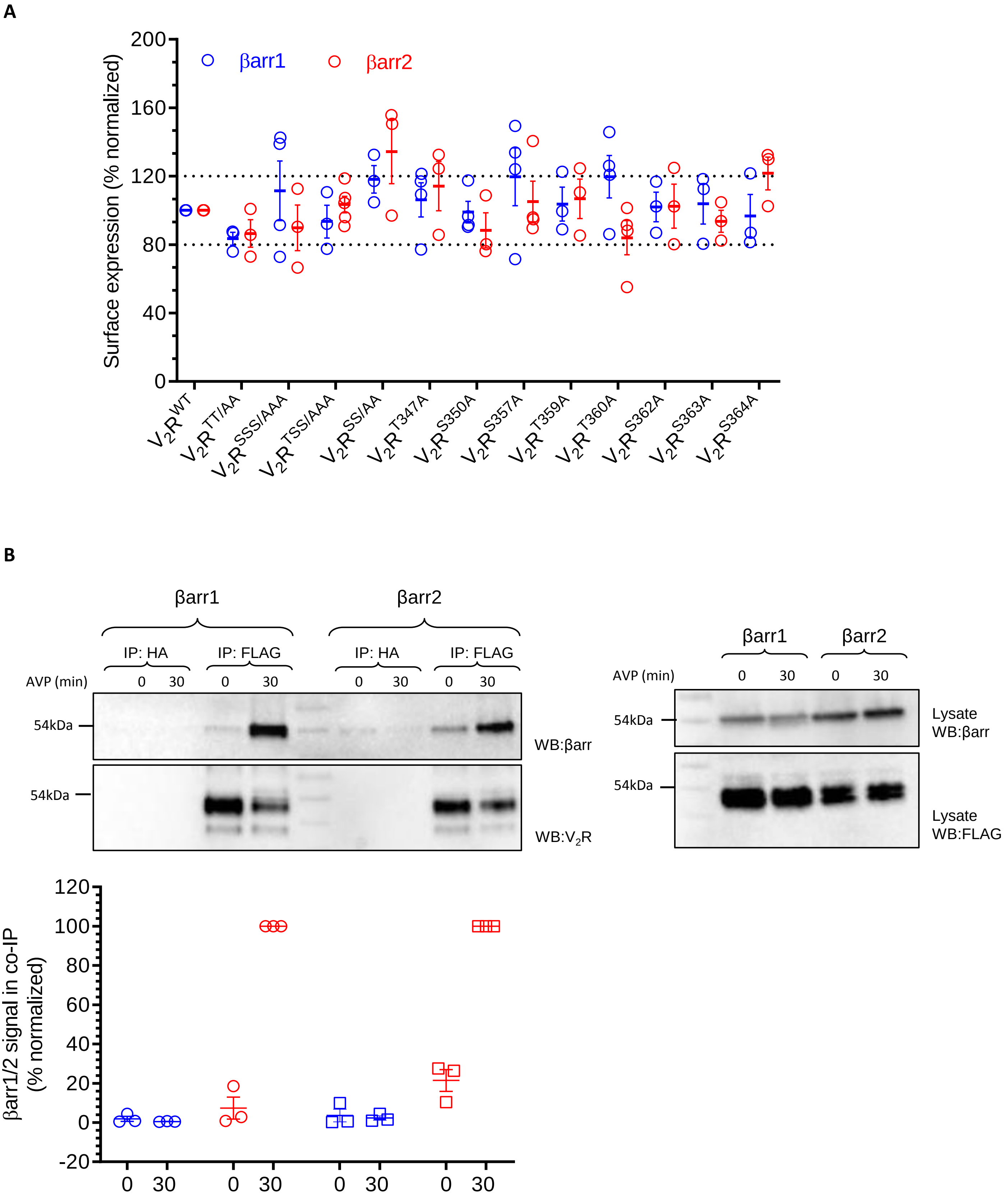
A
Surface expression (% normalized)
βarr1
βarr2
0
40
80
120
160
200
V2R WT
V2R TT/AA
V2R SSS/AAA
V2R TSS/AAA
V2R SS/AA
V2R T347A
V2R S350A
V2R S357A
V2R T359A
V2R T360A
V2R S362A
V2R S363A
V2R S364A
B
βarr1
βarr2
IP: HA
IP: FLAG
AVP (min)
0
30
54kDa
WB:βarr
WB:V2R
Lysate
WB:FLAG
βarr1/2 signal in co-IP (% normalized)
-20
100

Figure S1. Surface expression of V_2R mutants and specificity of co-immunoprecipitation assay. A. Surface expression of V_2R mutants as measured by whole-cell ELISA under co-expression conditions of βarr1 or 2. Values are normalized with respect to V_2R^{WT} treated as 100%, and represent mean±SEM from at least three independent experiments, each performed in duplicate. **B.** The specificity of our anti-Flag agarose based co-immunoprecipitation assay is established using anti-HA antibody agarose as a negative control on HEK-293 cells expressing Flag-tagged V_2R^{WT} and βarrs. Upon agonist-stimulation (100nM, AVP), we observe pull-down of βarrs with anti-Flag M2 antibody agarose through Flag-tagged V_2R but not with anti-HA antibody agarose. Cellular lysate taken before DSP cross-linking were probed for βarr1/2 and Flag-V_2R to verify comparable input in co-IPs. Representative images from three independent experiments, and densitometry-based quantification of data (mean±SEM), normalized with the signal at 30min agonist-stimulation for V_2R^{WT} (treated as 100%) are shown.

Figure S2

A

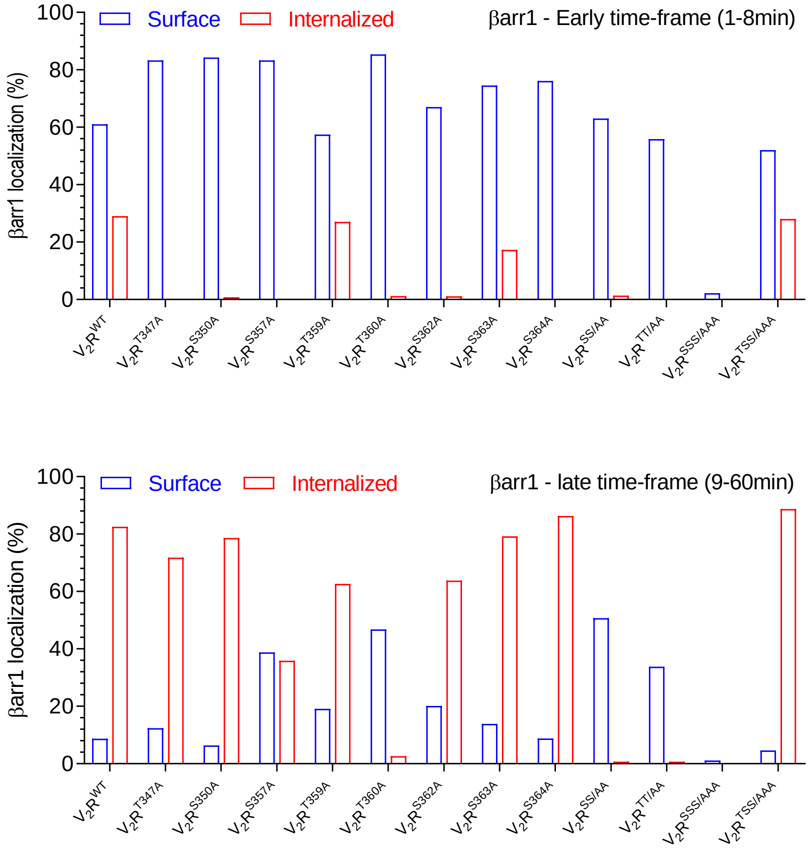
Surface
Internalized
βarr1 - Early time-frame (1-8min)
βarr1 localization (%)
0
20
40
60
80
100
V2RWT
V2RT347A
V2RS350A
V2RS357A
V2RT359A
V2RT360A
V2RS362A
V2RS363A
V2RS364A
V2RSS/AA
V2RTT/AA
V2RSSS/AAA
V2RTSS/AAA
βarr1 - late time-frame (9-60min)

B

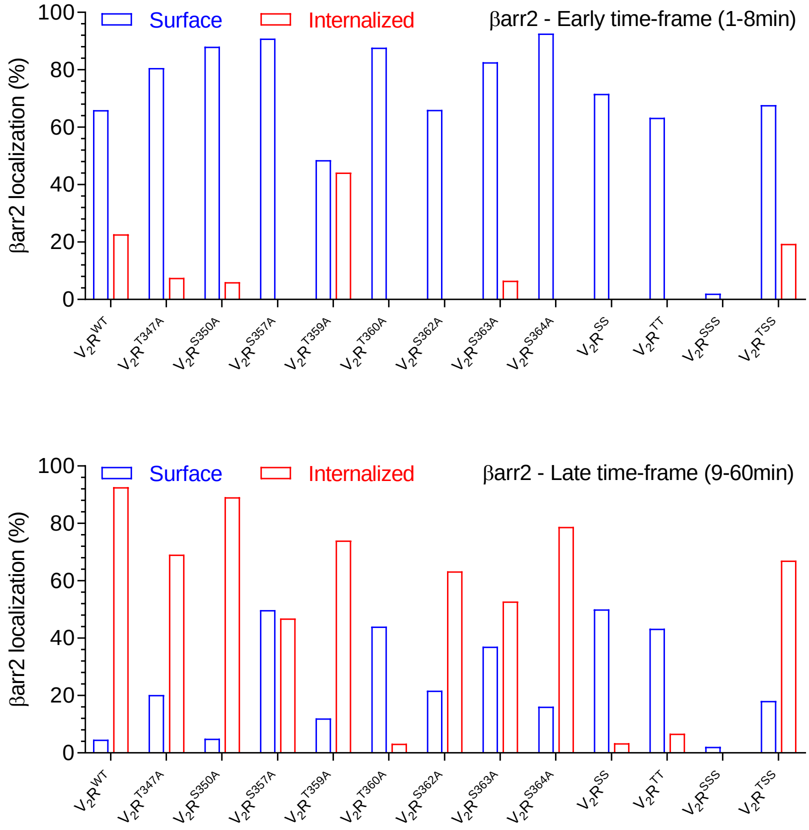
Surface
Internalized
βarr2 - Early time-frame (1-8min)
βarr2 localization (%)
0
20
40
60
80
100
V2RWT
V2RT347A
V2RS350A
V2RS357A
V2RT359A
V2RT360A
V2RS362A
V2RS363A
V2RS364A
V2RSS
V2RTT
V2RSSS
V2RTSS
βarr2 - Late time-frame (9-60min)

Figure S2. Quantification of agonist-induced βarr translocation as measured by confocal microscopy. A-B. Agonist-induced localization of βarrs for different V_2R mutants were measured by confocal microscopy as described earlier, and βarr localization in cells from multiple fields in at least three independent experiments were manually scored. Confocal images captured during 1-8min and 9-60min post-agonist stimulation were grouped under early and late time-frames, respectively. The localization of βarrs was scored as surface and internalized depending based on YFP fluorescence in the plasma membrane and punctate structures in the cytoplasm, respectively. Data are plotted as % of βarr localization pattern from more than hundred cells counted for each condition.

Figure S3

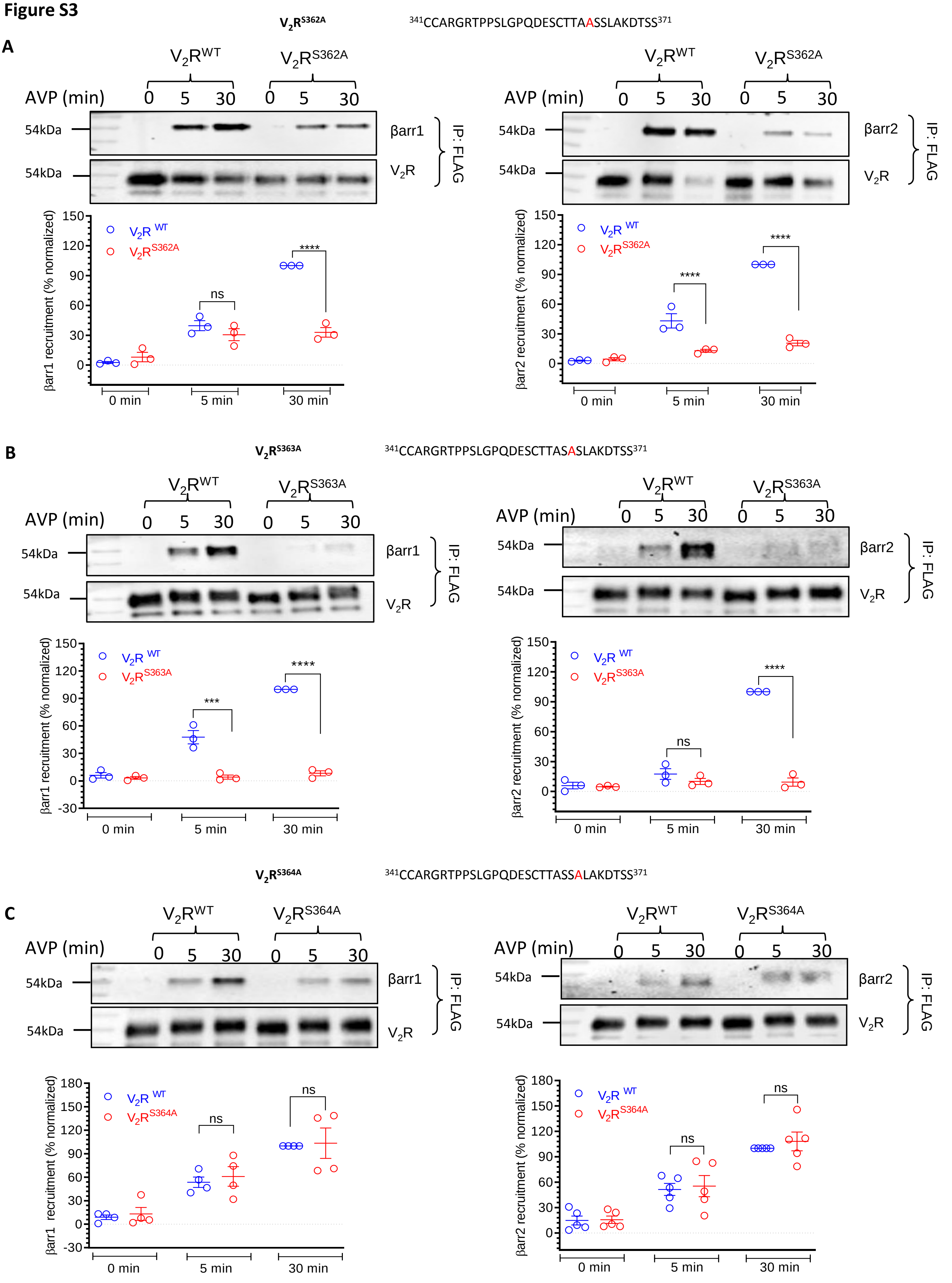
A
V2RS362A
341CCARGRTPPSLGPQDESCTTAASSLAKDTSS371
V2RWT
V2RS362A
AVP (min)
0 5 30 0 5 30
54kDa
βarr1
V2R
βarr2
IP: FLAG
βarr1 recruitment (% normalized)
βarr2 recruitment (% normalized)
V2R WT
V2RS362A
ns
****
0 min
5 min
30 min
B
V2RS363A
341CCARGRTPPSLGPQDESCTTASASLAKDTSS371
V2RS363A
***
C
V2RS364A
341CCARGRTPPSLGPQDESCTTASSALAKDTSS371
V2RS364A

Figure S3. Mutation of the individual sites in the SSS cluster differentially affects βarr recruitment. A-C. Mutation of S^{362} and S^{363} robustly inhibits agonist-induced (100nM AVP) βarr recruitment, however, S^{364} has a relatively smaller effect, as measured by co-immunoprecipitation (co-IP) experiment in HEK-293 cells. Representative images from three independent experiments (four for V_2R^{S364A}+βarr1 and five for V_2R^{S364A}+βarr2), and densitometry-based quantification of data (mean±SEM), normalized with the signal at 30min time-point for V_2R^{WT} is shown. Data are analyzed using Two-Way ANOVA (ns, non-significant; p<0.05; **p<0.01; ***p<0.001).

Figure S4

A

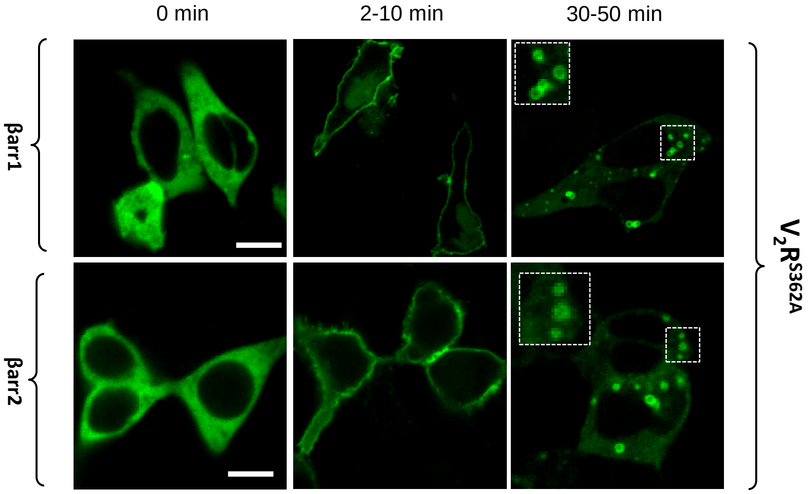

B

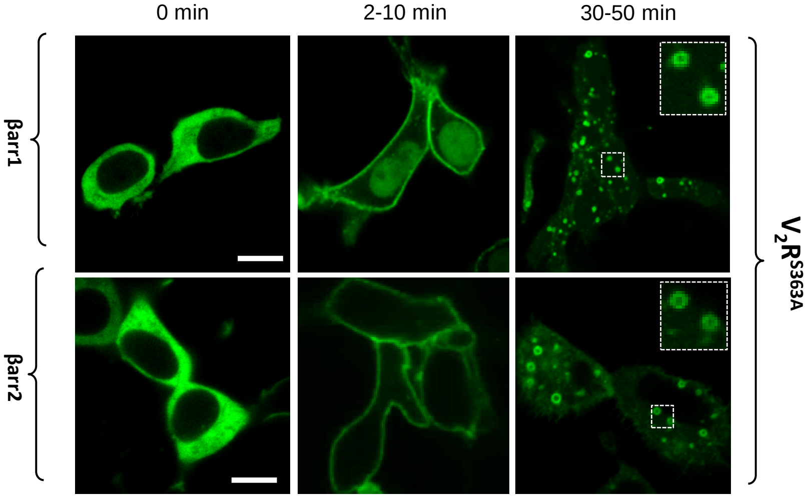

C

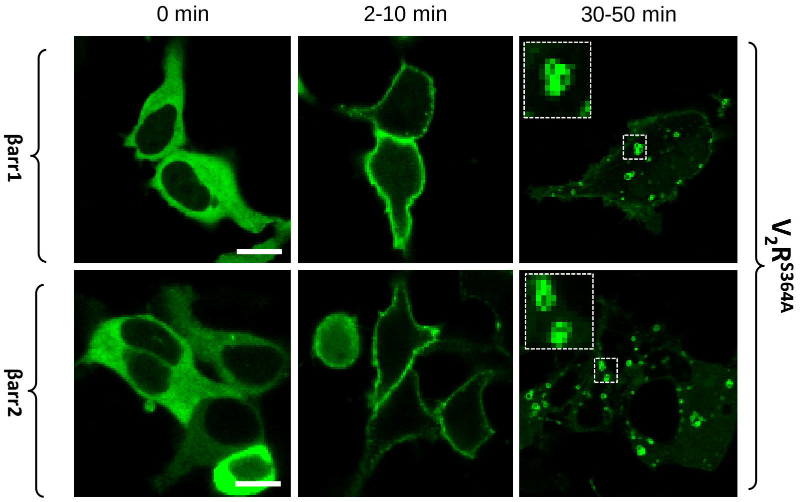

Figure S4. Mutation of the individual sites in the SSS cluster does not affect the overall pattern of βarr trafficking. A-C. Agonist-induced trafficking of βarrs for the V_2R^{S362A}, V_2R^{S363A} and V_2R^{S364A} are qualitatively similar to that of V_2R^{WT} as assessed by confocal microscopy in HEK-293 cells expressing the indicated receptor mutant and βarr-mYFP. Cells were stimulated with 100nM AVP and representative images from three independent experiments at indicated time-points are shown (scale bar = 10µm).

Figure S5

A

$V_2R^{SS/AA}$ [341]CCARGRTPPSLGPQDESCTTAAASLAKDTSS[371]

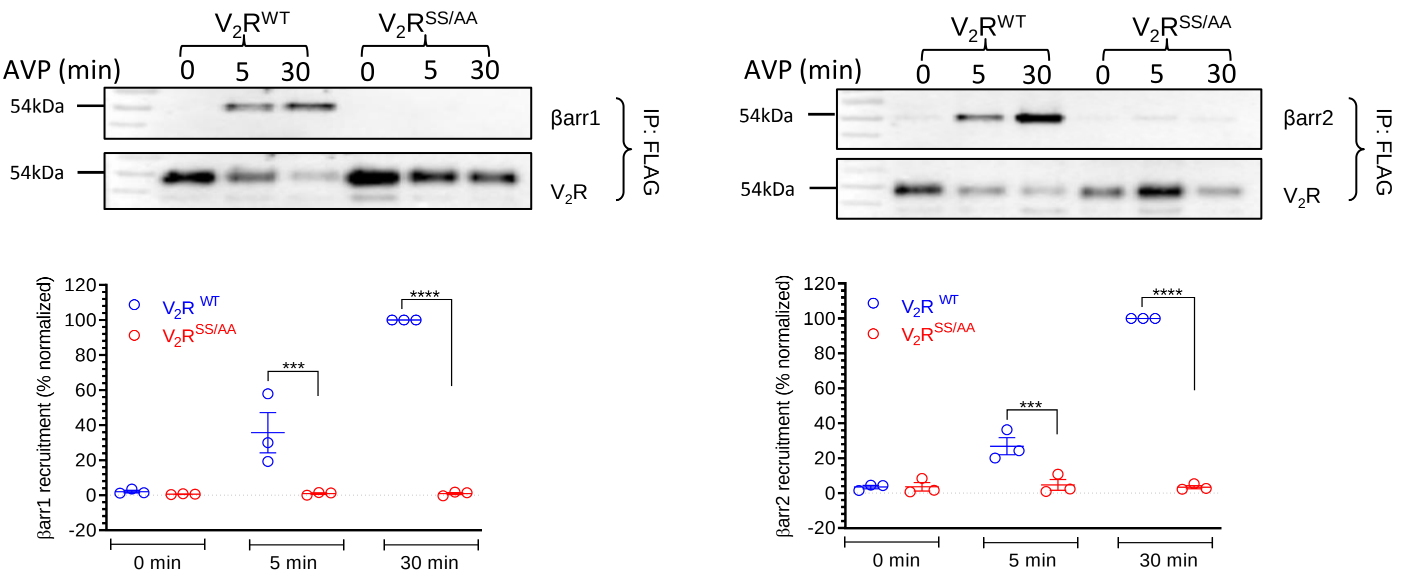

B

$V_2R^{SSS/AAA}$ [341]CCARGRTPPSLGPQDESCTTAAAALAKDTSS[371]

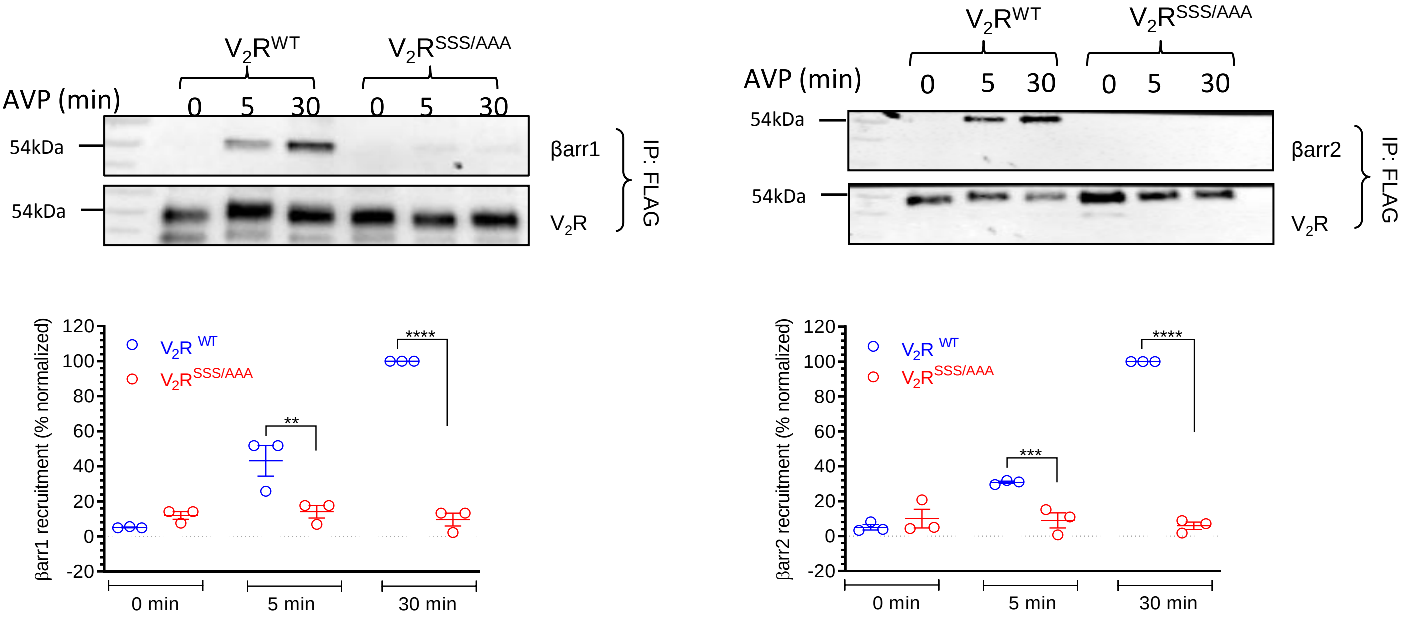

Figure S5. Double and triple phospho-site mutations in the SSS cluster ablate βarr recruitment. A-B. Mutation of S^{362} and S^{363} (i.e. $V_2R^{SS/AA}$) and S^{362}, S^{363} and S^{364} (i.e. $V_2R^{SSS/AAA}$) together nearly eliminate agonist-induced (100nM AVP) βarr recruitment as measured by co-immunoprecipitation (co-IP) experiment in HEK-293 cells. Representative images from three independent experiments, and densitometry-based quantification of data (mean±SEM), normalized with the signal at 30min time-point for V_2R^{WT} is shown. Data are analyzed using Two-Way ANOVA (**$p<0.01$; ***$p<0.001$; ****$p<0.0001$).

Figure S6

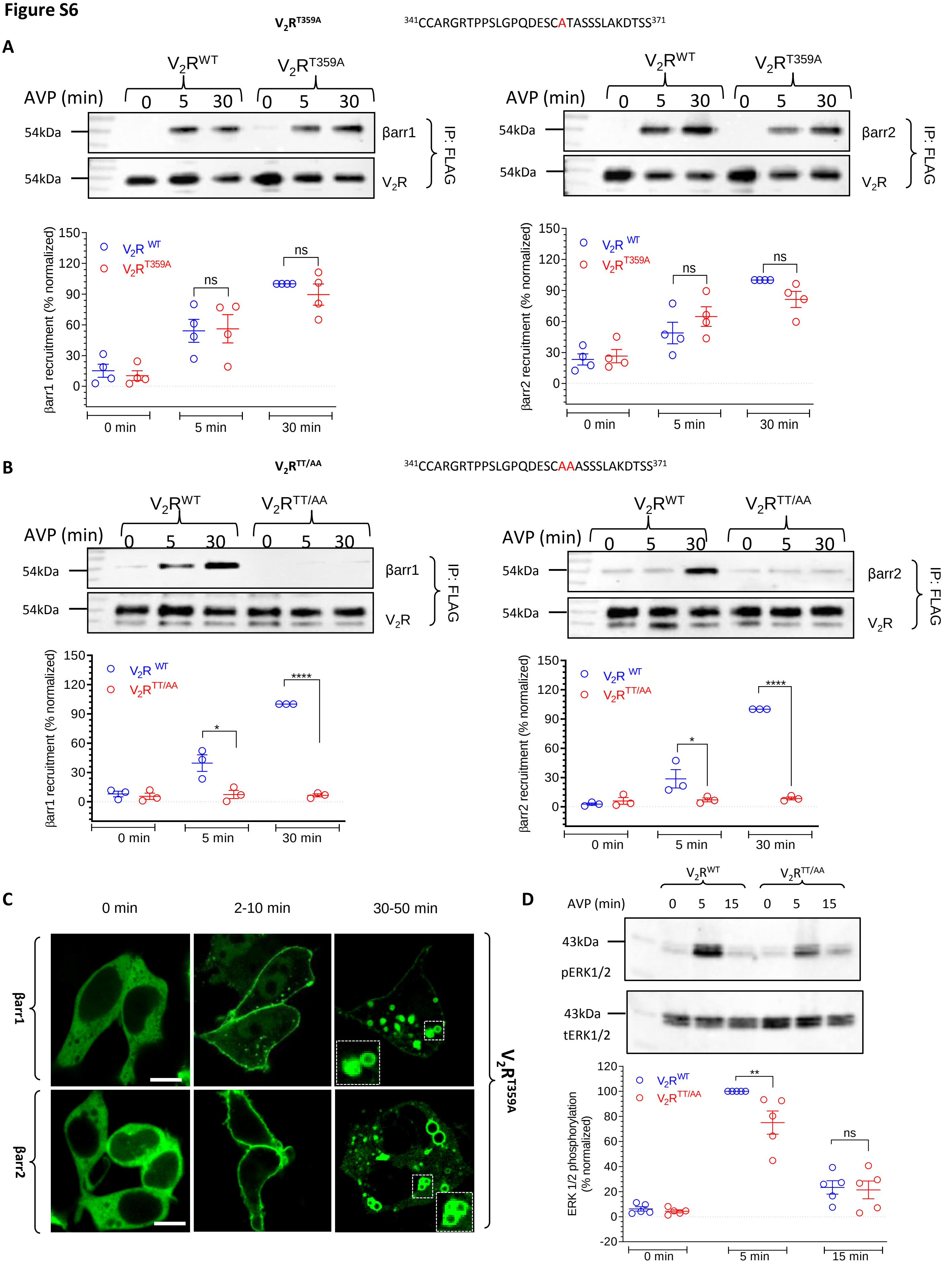

V2R T359A
341CCARGRTPPSLGPQDESCATASSSLAKDTSS371
A
V2R WT
V2R T359A
AVP (min)
0
5
30
54kDa
βarr1
βarr2
V2R
IP: FLAG
βarr1 recruitment (% normalized)
βarr2 recruitment (% normalized)
150
120
90
60
30
0
ns
0 min
5 min
30 min
V2R TT/AA
341CCARGRTPPSLGPQDESCAAASSSLAKDTSS371
B
*
****
C
0 min
2-10 min
30-50 min
βarr1
βarr2
D
AVP (min)
15
43kDa
pERK1/2
tERK1/2
ERK 1/2 phosphorylation (% normalized)
100
80
40
20
-20
**
15 min

Figure S6. T^{359} in the TT cluster is dispensable but double phospho-site mutation inhibits βarr recruitment. A-B. Mutation of T^{359} does not significantly influence but the double phospho-site mutation (i.e. T^{359}+T^{360}) nearly eliminates agonist-induced (100nM AVP) βarr recruitment as measured by co-immunoprecipitation (co-IP) experiment in HEK-293 cells. Representative images from four independent experiments (three for $V_2R^{TT/AA}$), and densitometry-based quantification of data (mean±SEM), normalized with the signal at 30min time-point for V_2R^{WT} is shown. Data are analyzed using Two-Way ANOVA (*p<0.01; ****p<0.0001). **C.** Mutation of T^{359} does not significantly alter agonist-induced βarr trafficking pattern as assessed by confocal microscopy in HEK-293 cells expressing the receptor mutant and βarr-mYFP. Cells were stimulated with 100nM AVP and representative images from three independent experiments at indicated time-points are shown (scale bar = 10μm). **D.** Double phospho-site mutation (i.e. T^{359}+T^{360}) results in a significant decrease in ERK1/2 activation, compared to V_2R^{WT}, at 5min after agonist-stimulation (100nM AVP). Representative images from five independent experiments, and densitometry-based quantification of data (mean±SEM), normalized with respect to the signal at 5min time-point for V_2R^{WT} (treated as 100%) is shown. Data are analyzed using Two-Way ANOVA (ns = non-significant; **p<0.01).

Figure S7

A

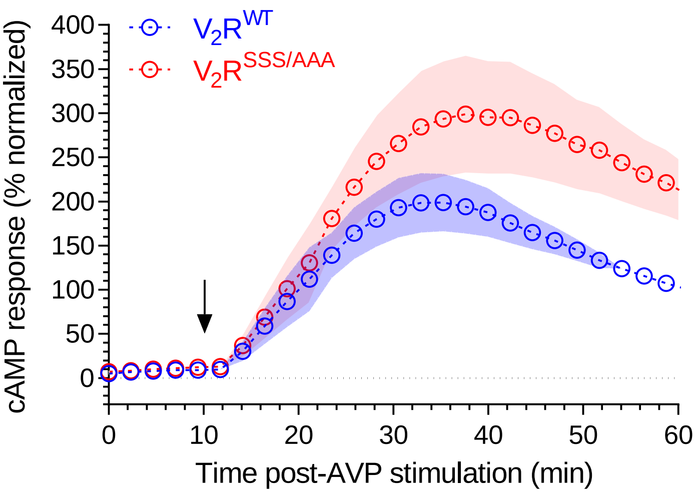

B

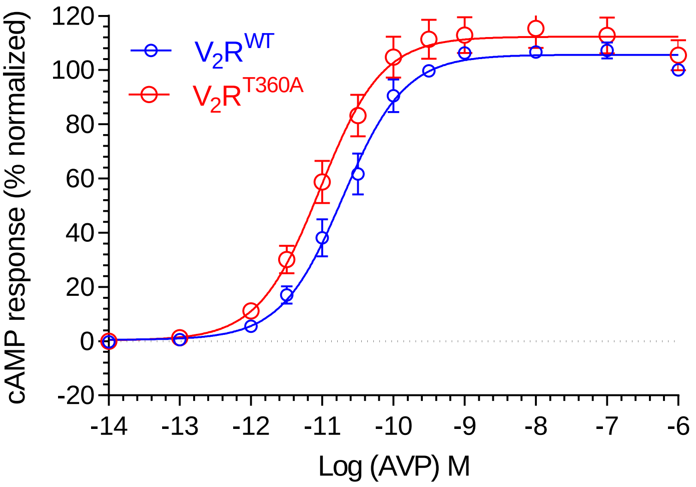

C

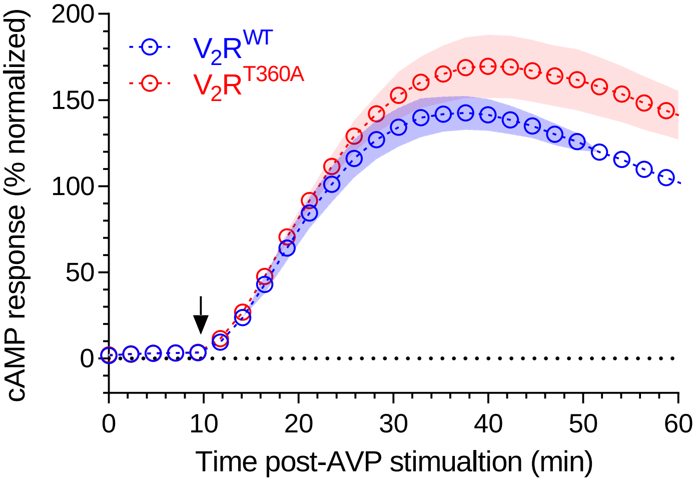

Figure S7. Agonist-induced cAMP response for the V_2R^{T360A} and $V_2R^{SSS/AAA}$ mutants. A. Time-course analysis of agonist-induced cAMP response for the V_2R^{WT} and $V_2R^{SSS/AAA}$ constructs at 10pM concentration of AVP reveals relatively sustained level of cAMP for the mutants as expected based on their lack of βarr recruitment. Data (mean±SEM) from six independent experiments (dose response presented in Figure 4F), each performed in duplicate and normalized with respect to the signal at 60min post-stimulation for V_2R^{WT}, are shown. **B-C.** Dose response and time-course analysis of agonist-induced cAMP response for V_2R^{T360A} as measured in HEK-293 cells using the GloSensor assay. Data (mean±SEM) from four independent experiments, each performed in duplicate, are presented here. Data are normalized with respect to the response at 1μM concentration of AVP for V_2R^{WT} (treated as 100%) for the dose response curve, and with respect to the signal at 60min post-stimulation for V_2R^{WT} for the time-course analysis. The arrows in panels A and C indicate the addition of agonist.

Figure S8

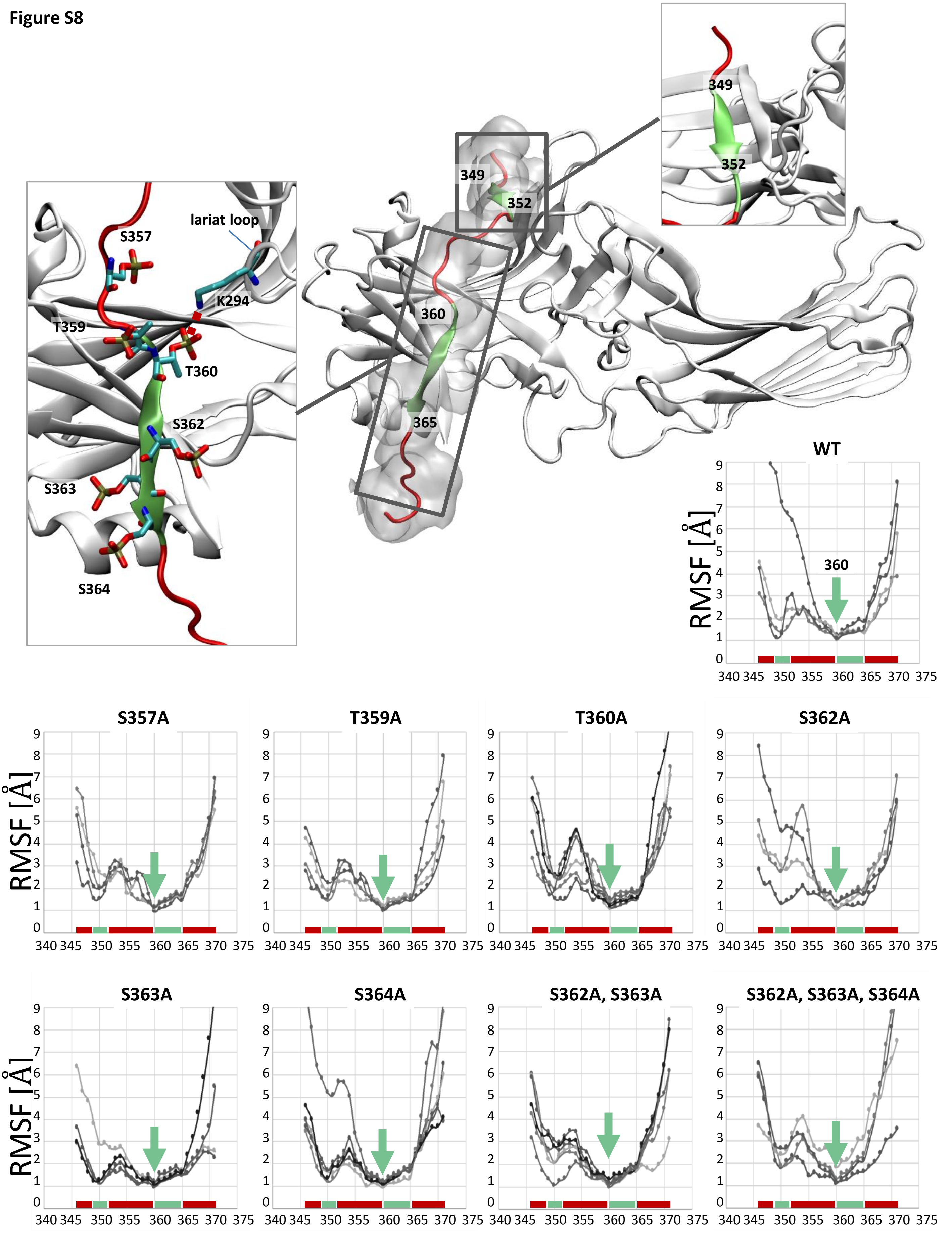
lariat loop
S357
K294
T359
T360
S362
S363
S364
349
352
360
365
WT
S357A
T359A
T360A
S362A
S363A
S364A
S362A, S363A
S362A, S363A, S364A
RMSF [Å]
340 345 350 355 360 365 370 375

Figure S8. Binding profile of the V_2Rpp and mutated versions to βarr1. Classical unbiased simulation was used to monitor the dynamics of the V_2Rpp and the phospho-site mutants investigated in this study. A quantitative measure of V_2Rpp dynamics is obtained by computing root mean square fluctuation (RMSF) per residue. We find that the V_2Rpp as well as the studied mutants show a similar fluctuation profile (see RMSF plots). Not surprisingly, more fluctuation (i.e. higher RMSF) is found for the N- (346 to 348) and C-terminal ends (366 to 372). In contrast, stable fragments are found at distinct sections of the V_2Rpp, namely residue 349 to 352 and 360 to 365 (i.e. low RSMF). Note, both sections adopt an extended β-strand that pack against existing strands in the βarr1 via backbone-backbone interactions (indicated in green in the panel above). This secondary structural arrangement is the reason for stability and low RMSF values in these positions. We observed that the most stable residue is consistently observed at T^{360} for all simulated systems (highlighted with a green arrow in plots). T^{360} is part of a β-strand and additionally interacts with K^{294} in the lariat loop via a strong electrostatic interaction (top left). Interestingly, we observed the same stability pattern even for the T^{360}A mutant which we ascribe to its backbone-backbone interaction with βarr1.
